# Ionic basis of atrioventricular conduction: ion channel expression and sarcolemmal ion currents of the atrioventricular canal of the rainbow trout (*Oncorhynchus mykiss*) heart

**DOI:** 10.1007/s00360-021-01344-2

**Published:** 2021-02-11

**Authors:** Minna Hassinen, Irina Dzhumaniiazova, Denis V. Abramochkin, Matti Vornanen

**Affiliations:** 1 Department of Environmental and Biological Sciences, University of Eastern Finland, P.O. Box 111, 80101 Joensuu, Finland; 2 Department of Human and Animal Physiology, Lomonosov Moscow State University, Leninskiye Gory, 1, 12, Moscow, Russia; 3 Laboratory of Cardiac Electrophysiology, National Medical Research Center for Cardiology, Moscow, Russia; 4 Department of Physiology, Pirogov Russian National Research Medical University, Moscow, Russia

**Keywords:** Fish heart, Ion channel transcripts, Ion current densities, Electrical excitability, Atrioventricular nodal cells

## Abstract

Atrioventricular (AV) nodal tissue synchronizes activities of atria and ventricles of the vertebrate heart and is also a potential site of cardiac arrhythmia, e.g., under acute heat stress. Since ion channel composition and ion currents of the fish AV canal have not been previously studied, we measured major cation currents and transcript expression of ion channels in rainbow trout (*Oncorhynchus mykiss*) AV tissue. Both ion current densities and expression of ion channel transcripts indicate that the fish AV canal has a characteristic electrophysiological phenotype that differs from those of sinoatrial tissue, atrium and ventricle. Two types of cardiomyocytes were distinguished electrophysiologically in trout AV nodal tissue: the one (transitional cell) is functionally intermediate between working atrial/ventricular myocytes and the other (AV nodal cell) has a less negative resting membrane potential than atrial and ventricular myocytes and is a more similar to the sinoatrial nodal cells in ion channel composition. The AV nodal cells are characterized by a small or non-existent inward rectifier potassium current (*I*_K1_), low density of fast sodium current (*I*_Na_) and relatively high expression of T-type calcium channels (CACNA3.1). Pacemaker channel (HCN4 and HCN2) transcripts were expressed in the AV nodal tissue but *I*_f_ current was not found in enzymatically isolated nodal myocytes. The electrophysiological properties of the rainbow trout nodal cells are appropriate for a slow rate of action potential conduction (small *I*_Na_) and a moderate propensity for pacemaking activity (absence of *I*_K1_).

## Introduction

The activity of the vertebrate heart is controlled by rhythmic electrical impulses (action potentials, APs), which originate from the sinoatrial (SA) pacemaker. APs propagate at alternating velocity through the histologically and functionally differentiated sections of the heart (Irisawa 1978; Stoyek et al. 2016). The rhythmic propagation pattern of electrical excitation (EE) is necessary for the synchrony of atrial and ventricular contractions and effective pump function of the heart (Billette and Tadros 2019).

The heart of teleost fishes consists of six consecutive histologically identifiable parts: *Sinus venosus*, atrium, atrioventricular (AV) canal, ventricle, *conus arteriosus* and *bulbus arteriosus* in the order from caudal to cranial direction (Icardo 2017). In fish hearts, the annular SA pacemaker at the junction between the *sinus venosus* and the atrium is the site of origin for EE (Yamauchi and Burnstock 1968; Haverinen and Vornanen 2007). From there, APs proceed first to the atrium and via the AV canal to the ventricle triggering sequential contractions of these two muscular chambers of the fish heart (McWilliam 1885; Keith and Flack 1907; Nair 1976; Arbel et al. 1977; Sedmera et al. 2003). In the atrial wall, the propagation of AP is fast, but it slows down sharply in the AV canal. This creates a delay that allows enough time for ventricular filling before the ventricle contracts (Meijler and Janse 1988; Sedmera et al. 2003; Milan et al. 2006; Haverinen et al. 2014; Stoyek et al. 2016; Billette and Tadros 2019). Another inherent characteristic of the vertebrate AV tissue is its ability to block AP conduction if the frequency of APs arriving from the atrial myocardium is high (Irisawa 1978; Billette and Tadros 2019). This prevents atrial tachycardia from triggering life-threatening arrhythmias in the ventricle(s). Finally, the AV nodal tissue can generate spontaneous APs and act as an auxiliary pacemaker if the SA pacemaker happens to fail (Meijler and Janse 1988a; Stoyek et al. 2016; Billette and Tadros 2019). When the slowly propagating impulse of the AV canal arrives to the ventricle, its rate accelerates again and induces a fast contraction of the ventricular myocardium (Sedmera et al. 2003). It is noteworthy that there is no histologically identifiable conductance pathway in the ventricle of the fish heart that is similar to the His–Purkinje system of the endothermic animals (Solc 2007).

The AV canal of the fish heart is homologous to the mammalian AV node (AVN) and is the sole conductance pathway for EE between the atrium and the ventricle (Solc 2007; Icardo and Colvee 2011; Jensen et al. 2012). It is a ring- or tube-shaped segment formed by myocardial and connective tissues to which the AV valves are attached (Santer and Cobb 1972; Icardo and Colvee 2011). The electric activity of the fish AV canal is poorly known and to our knowledge ion channel composition and ion currents of the fish AV cells have not been previously studied. This is a serious deficiency, because AV block is a common malfunction of the fish heart under different stresses and in response to environmental toxicants and medicinal drugs (McWilliam 1885; Arbel et al. 1977; Sarmah and Marrs 2016; Vornanen 2017; Cassar et al. 2019; Monteiro et al. 2020). In particular, it is known that at critically high temperatures, cardiac output in fish is compromised due to the functional AV block (Vornanen et al. 2014; Badr et al. 2016; Haverinen and Vornanen 2020). Furthermore, understanding the function of the fish AV canal is vital for the use of zebrafish as an animal model of the human heart. In particular, because zebrafish is suggested as a useful translational model for preclinical screening of cardiovascular drug molecules and safety pharmacology of environmental toxicants (Barros et al. 2008). To this end, our objective was to characterize the ion (Na^+^, K^+^, Ca^2+^) channel composition of the fish AV tissue at transcript level and to measure the density of major sarcolemmal ion (Na^+^, K^+^, Ca^2+^) currents in different cell types of the AV canal. We used quantitative PCR and single cell patch clamp to quantitate ion channel expression and ion current densities, respectively, in the AV canal of the rainbow trout (*Oncorhynchus mykiss*).

## Materials and methods

### Animals

Rainbow trout (*Oncorhynchus mykiss*) were obtained from a local fish farm (Kontiolahti, Finland). Fish with two different sizes were used: smaller ones for the histological staining (28.08 ± 5.63 g, *n* = 6) to allow efficient penetration of fixative to the heart, and bigger fish (299.68 ± 49.79 g) for the quantitative PCR (*n* = 5) and electrophysiological recordings (*n* = 8) to make it easier to obtain enough material from the small SA and AV regions. In the laboratory, the fish were reared in 500-L stainless steel tanks at + 12 °C in 12:12 h light:dark photoperiod and fed five times per week commercial fish food (Ewos, Turku Finland). All experiments were made with the consent of the national committee for animal experimentation (permission ESAVI/8877/2019).

### Histology

Fish was stunned by a quick blow to the head and killed by cutting the spine immediately behind the head. The heart was dissected and rinsed in phosphate-buffered saline before fixation in Bouin’s fixative (RAL Diagnostics, Martillac, France) for at least 24 h. The hearts were dehydrated in graded series of ethanol, cleared in xylene, and embedded in UltraPar paraffin (J.T.Baker, Deventer, Holland). Sections (7 µm) were cut using Leica RM2165 microtome (Leica Microsystems, Nußloch, Germany), attached on SuperFrost Plus slides (VWR International, Leuven, Belgium) and stained with Masson’s trichrome. Stained sections were photographed using Leica DMi1 (Leica Microsystems, Wetzlar, Germany).

### Whole-cell patch clamp

For the isolation of myocytes of AV canal, ventricle and atrium, the cannula was inserted in the ventricle through the aortic bulb and the heart was retrogradely perfused for 10–12 min with proteolytic enzymes (collagenase type 1A, 0.75 mg ml^−1^; trypsin type IX, 0.5 mg ml^−1^ both from Sigma and fatty acid free bovine serum albumin from Serva, 0.75 mg ml^−1^) as previously described in detail (Vornanen 1997; Haverinen and Vornanen 2007). Immediately upon completion of the perfusion, the AV region was excised, minced, and incubated in the enzyme solution for additional 20 min. After the enzymatic digestion, the tissue was triturated with a Pasteur pipette to release single myocytes. Cells were stored up to 8 h at + 5 °C in the cardioplegic low-Na^+^ solution containing (mmol L^−1^) NaCl 100, KCl 10, KH_2_PO_4_·2H_2_O 1.2, MgSO_4_·7H_2_O 4, taurine 50, glucose 10 and Hepes 10 at pH of 6.9. Ventricular and atrial myocytes were released from the tissue by mincing and triturating of ventricular and atrial muscle, respectively, immediately after the 10–12 min whole heart perfusion. Atrial and ventricular myocytes were used for comparison with AV canal myocytes.

A small aliquot of myocyte suspension was placed in the experimental chamber (RCP-10 T, Dagan, Maryland, MI, USA, volume 150 µL) and superfused at the rate of about 1.5 ml min^−1^ with the external K^+^-based physiological saline solution containing (in mmol L^−1^) NaCl 150, KCl 5.4, NaH_2_PO_4_ 0.4, MgSO_4_ 1.5, CaCl_2_ 1.8, glucose 10 and Hepes 10 at pH 7.7 (adjusted with KOH). Temperature of the external solution was regulated to + 12 ℃ using a Peltier device (TC-100, Dagan, Maryland, MI, USA). Ionic currents were recorded in the voltage clamp mode of the whole-cell patch clamp technique using the Axopatch 1-D amplifier (Molecular Devices, CA, USA) and the pClamp 8.2 software package. Patch pipettes of 1.5–2.5 MΩ resistance were pulled from borosilicate glass (Hilgenberg GmbH, Germany). Pipette capacitance, access resistance and whole cell capacitance were routinely cancelled.

For recording of K^+^ currents, the pipettes were filled with K^+^-based electrode solution containing (in mmol l^−1^): 140 KCl, 1 MgCl_2_, 5 EGTA, 4 MgATP, 0.3 Na_2_GTP and 10 HEPES with pH adjusted to 7.2 with KOH (at + 20 ℃). The standard external K^+^-based physiological solution was used. Similar conditions were used for measurements of membrane potential in the current clamp mode. For recording of Ca^2+^ currents, KCl in both external and pipette solutions was substituted with equimolar concentration of CsCl. The external solution for I_Na_ recordings contained less Na^+^ to reduce the driving force for Na^+^ influx (in mmol l^−1^): 20 NaCl, 120 CsCl, 1 MgCl_2_, 0.5 CaCl_2_, 10 glucose, 10 HEPES, pH adjusted to 7.7 with CsOH. The pipette solution for I_Na_ contained (in mmol l^−1^): 5 NaCl, 130 CsCl, 1 MgCl_2_, 5 EGTA, 5 Mg_2_ATP, 5 HEPES, pH adjusted to 7.2 with CsOH (Vornanen et al. 2011).

### Quantitative PCR

Transcripts (mRNA) of 41 ion channel genes and 1 transcription factor were measured from 5 different tissue sections of the trout heart. To this end, the heart was excised and placed in cold physiological saline solution. A thin (about 2 mm in width) tissue ring was cut out from the atrioventricular junction so that the whole AV canal was included. This piece of tissue was divided in the middle into two parts, the atrial side (AV-atr) and the ventricular (AV-ven) side. This was done, because the fish AV canal is not a homogenous and sharply delimited structure, which could be dissected clean from the surrounding atrial and ventricular muscles. Gene expression was separately measured for AV-atr and AV-ven. In addition to AV-atr and AV-ven, gene expression was measured from atrial and ventricular tissue and SA pacemaker. The fish cardiac pacemaker (hereafter called SA node or SAN) is an annular tissue mass at the border between *sinus venosus* and the atrium. SAN was obtained as a thin ring of tissue from this border zone. Tissue pieces (SAN, atrium, AV-atr, AV-ven and ventricle) were quickly frozen in liquid nitrogen and stored at − 80 °C until used in experiments. Total RNA was extracted by TRI Reagent Solution (Thermo Scientific, Vilnius, Lithuania) and stored at − 80 °C. The integrity and quantity of RNA was assessed by agarose gel electrophoresis and NanoDrop ND-1000 Spectrophotometer (NanoDrop Technologies, Wilmington, DE, USA), respectively. An aliquot of 1.5 µg of total RNA was treated with RNase-free DNase (Thermo Scientific) and converted to cDNA by Maxima H Minus First Strand cDNA Synthesis Kit (Thermo Scientific) using a mixture of random hexamers and oligo(dT)_18_ primers. From each DNase-treated sample, a control reaction containing all other reaction components except RT enzyme was produced. Primer pairs were designed using Primer3 software (http://bioinfo.ut.ee/primer3-0.4.0/primer3/). All primer pairs were tested using rainbow trout genomic DNA as the template, and only primers giving 95–105% efficacy were chosen. Amplification was performed as triplicates from each sample using Maxima SYBR Green qPCR Master Mix (Thermo Scientific), primers presented in Table 1 and AriaMx Real-Time PCR System (Agilent Technologies Inc., Santa Clara, CA, USA) with the following cycling conditions: initial denaturation and enzyme activation at + 95 °C for 10 min followed by 40 cycles of denaturation at + 95 °C for 15 s, annealing at + 57 to 58 °C for 30 s, and extension at + 72 °C for 30 s. After amplification, a melting curve analysis was performed by rising the temperature from + 65 to + 95 °C and reading the fluorescence after every 0.5 ℃ increase to check the specificity of amplification. A couple of qPCR products amplified with each primer pair was run on an agarose gel to check the length and specificity of the amplification. Comparative quantification was used to calculate the mRNA level of the genes of interest. The mRNA expression of the studied genes was normalized to the transcript abundance of reference gene DnaJA2 using ΔCt method (Vornanen et al. 2005; Hassinen et al. 2015).

**Table 1:** Accession numbers (GenBank) and primers for the genes studied in qPCR. Amplicon length, melting temperature of the product and annealing temperature used in qPCR are represented

Gene	Protein	Accession no	Primers (5′-3′)	Product (bp)	Tm (°C)	Annealing temp. (°C)
CACNA1C	Cav1.2	XM_021595819	F: tgacatgaccatcgaggaga R: ctgttgcagtgcatgtaggg	105	82.0	58
CACNA1Daa	Cav1.3aa	AF450282	F: cctgcataggagtacagctct R: gcctctctctcttttggatggc	144	74.5	57
CACNA1Dab	Cav1.3ab	XM_021567482	F: ctgcataggggtgcagctc R: gccacactctcttatggacgaa	143	83.0	58
CACNA1Dba	Cav1.3ba	XM_021614407	F: ttcgctaccctaccgttcgt R: ttgacactccttacgggaca	147	81.5	58
CACNA1Dbb	Cav1.3bb	XM_021564469	F: ttctgtacccaaccatccgc R: ttgaaactccctatggggca	147	80.5	58
CACNA1Ga	Cav3.1a	XM_021575364	F: cgtcgagctcggcaactat R: ggacttcatgtccatctgacac	132	83.5	58
CACNA1Gb	Cav3.1b	XM_021567299	F: gagaaagagtaggaagacttcgg R: tctcacactcccgttcactaa	130	78.5	58
CACNA1Ha	Cav3.2a	XM_021592899	F: ccgtctgtaacaggggagt R: gtcctcctccatgtcatcgt	106	80.5	58
CACNA1Hb	Cav3.2b	XM_021593501	F: gcggaggagtcaatcaagtg R: tctgattggtccaggaggtc	120	82.5	58
CACNA1Ia	Cav3.3a	XM_021625686	F: agtgactcccagggttcaga R: cctcctcggctactgaactg	109	84.5	58
CACNA1Ib	Cav3.3b	XM_021570408	F: ccgtagtggatcacagtgga R: cctgtgcaggtgagtacagc	102	82.0	58
HCN1a	HCN1a	NM_001124318	F: cagccacctctttgacctaca R: agttcaggttgccgtggat	129	87.5	58
HCN1b	HCN1b	XM_021603242	F: agtcatccgcccatttaacatg R: gagacactggggacagggta	115	86.0	58
HCN2aa	HCN2aa	XM_021588920	F: cgtgtggctttcgcctcta R: ggctatcgatcaaatccttgttgg	118	86.5	57
HCN2ab	HCN2ab	XM_021614280	F: cctctggcgcatccagga R: gcgagtgggtgcttttgtgt	142	86.5	58
HCN2ba	HCN2ba	XM_021589263	F: actgttacatcgctggggatg R: ggaggctggctataaactcactc	112	84.0	58
HCN2bb	HCN2bb	XM_021601253	F: gggacaggcatgggtgc R: tggtcggctttggagtccttt	113	84.5	58
HCN3a	HCN3a	XM_021571553	F: atggaggcttcggatgtgta R: ctgatggatgtagcggatgaag	116	84.5	57
HCN3b	HCN3b	XM_021619039 XM_021598239	F: ggctggactcagaggtttaca R: ctggtggatgtagcgtatgagt	115	84.5	57
HCN4a	HCN4a	XM_021607247 XM_021585508	F: agctactggtggagcaatgg R: gggggctgatactacaacca	125	81.0	58
HCN4ba	HCN4ba	XM_021580609	F: ctgtccctacacgaccacaa R: acctacaacgcctccaacac	127	83.0	58
HCN4bb	HCN4bb	XM_021601191	F: tccagaccgtctctctccat R: gaggtgtgacgtgggagttt	104	80.0	58
ISL1 ISL1L	ISL1 ISL1L	XM_021622253 XM_021605098	F: aaaccagatccacgaccagt R: cagtaggttttcccgtctcg	138	82.0	58
KCNH2aa	Kv11.1aa	XM_021589368	F: agtttgaggggctgaggaa R: agcactgctgatgttgagga	143	80.5	58
KCNH2ab	Kv11.1ab	XM_021613401	F: gcctttccaagtcctgtgag R: aggaggagtcttggggagag	113	82.0	58
KCNH2ba	Kv11.1ba	XM_021622309	F: acaagtatgtcacagccctctat R: gagcatgacacagatggagaat	112	80.5	58
KCNH2bb	Kv11.1bb	XM_021562719	F: agtacgtgacggcactctac R: gcatgacgcagatggaaaag	108	81.5	58
KCNH6a	Kv11.2a	XM_021558455	F: ttcctgctcaacgagatgga R: tggtccggaagttgatgaga	127	83.5	58
KCNH6b	Kv11.2b	XM_021567337	F: ttcctattggatgagcgtgg R: gtgcgaaagttgatcccgata	125	82.5	58
KCNH7aa	Kv11.3aa	XM_021571238	F: taagcagtccctcccacaag R: ccgcgtgtcattggtgta	142	88.0	58
KCNH7ab	Kv11.3ab	XM_021608684	F: ttactacagagtcaatgcggg R: cctcttagctcttccgacct	107	78.0	58
KCNH7ba	Kv11.3ba	XM_021597081	F: gtatggagctgctgtgctaatg R: tcttgccaatggagacaccc	155	81.5	58
KCNH7bb	Kv11.3bb	XM_021579952	F: cggagcggctgtactcatc R: tcttgccaatggatacccca	152	82.0	58
KCNJ2a	Kir2.1a	XM_021576592 XM_021581081	F: ggcgctgactaacaaagagga R: tctagtggcacagtggcctg	117	83.0	58
KCNJ2b	Kir2.1b	XM_021559076	F: gaggtggcccttgaaaaagt R: tgttgcgttctcaatgtcgttc	129	80.0	58
KCNJ12a	Kir2.2a	XM_021565106 XM_021575489	F: acctgtacaaggtggattactc R: acagaaggagttggaggtgg	122	81.5	58
KCNJ12b	Kir2.2b	XM_021599878 XM_021624360	F: gccagtacaaagtggattacg R: taggatccttgtcctcgtcc	111	82.0	58
KCNJ14	Kir2.4	XM_021622272	F: tgctacgagaacgaagtagcc R: ttagtgtgggtgggttgtga	122	85.0	58
SCN4Aa	Nav1.4a	XM_021565351	F: cgctccaagaggcagataag R: acccagccaattccatctct	130	83.5	57
SCN4Aba	Nav1.4ba	EF203231	F: tcagctggttgggatggtc R: aacaaattcccaaaccaggg	127	82.0	57
SCN4Abb	Nav1.4bb	XM_021624591	F: ttagccggttgggacagtg R: agaattccccaaccaaggct	125	81.5	57
SCN5LAa	Nav1.5a	XM_021614310	F: tcatcatccagcgctgttac R: tgcaagttcaggttggtctg	103	80.5	57
SCN5LAba	Nav1.5ba	XM_021562569	F: tggatgctctcaaacagcag R: cgacacatcctcctgcttg	110	81.5	57
SCN5LAbb	Nav1.5bb	XM_021622315	F: ggacatgtccgcagcagtc R: cctcttgtccgtggagcttt	127	83.0	57
DnaJA2	DnaJA2	XM_021585925	F: ttgtaatggagaaggtgagg R: tgggccgctctcttgtatgt	231	83.5	57–58

Gene paralogues are named as -a and -b. If more than two paralogues exist for the same gene, paralogues are named as -aa, -ab, -ba and –bb

### Statistics

The results are represented as means ± s.e.m. Normality of distribution was tested using Shapiro–Wilk test and if variables were not normally distributed, logarithmic transformations were made to get the data normally distributed before statistical testing. Equality of variances was checked and comparisons between mRNA amounts were performed using one-way ANOVA followed with paired comparisons using Tukey’s (equal variances) or Dunnett’s T3 (unequal variances) post hoc tests. The densities of ionic currents were compared using two-way ANOVA with Tukey’s multiple comparisons test. Student’s *t* test was used to compare the capacitances of nodal and transitional cells. A *p *value < 0.05 was deemed to be statistically significant.

## Results

### Histology of AV canal and AV myocytes

**Fig. 1:**
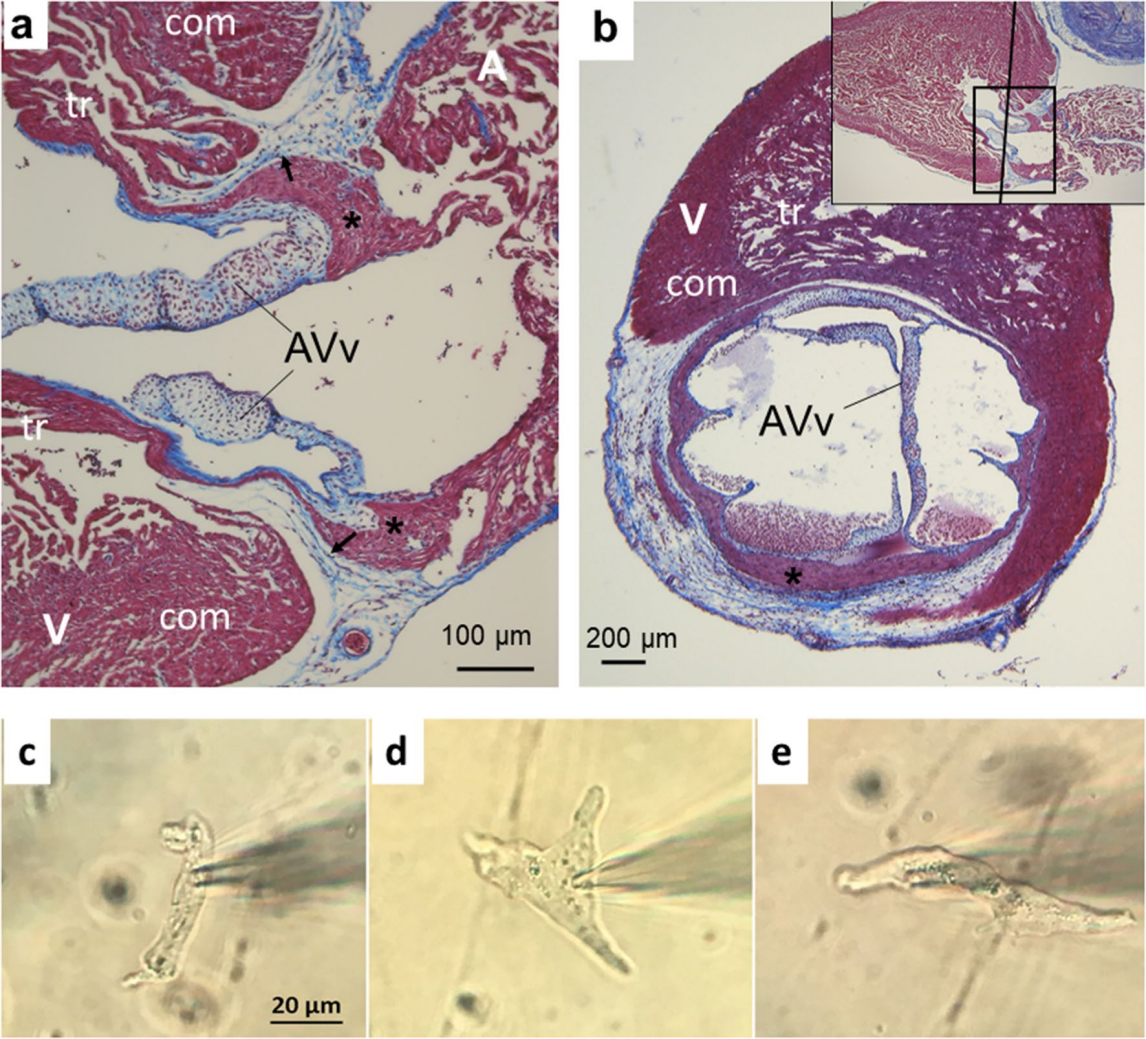
Histological characteristics of AV canal and AV cell types of rainbow trout heart. **a**, **b** Tissue sections stained with Masson’s trichrome. **a** Longitudinal section shows that the AV region is formed by a compact myocardium. It is continuous with the atrial muscle, surrounded laterally on both sides by connective tissue, and extends to endocardial trabeculae of the ventricle as a thin layer. The arrows indicate that the AV tissue is isolated from the compact ventricular myocardium by connective tissue. **b** Transverse section shows that the AV muscle forms a thin ring or tube that is surrounded on both sides by connective tissue. The inset in the upper corner shows the enlarged tissue area in the (**a**) and the sectioning level of the tissue in the (**b**). *A* atrium, *AVv* atrioventricular valves, *V* ventricle; com, compact myocardium, *tr* endocardial trabeculae, *AV myocardium; arrowhead, connective tissue. **c**, **d** Photomicrographs of morphological cell types of the rainbow trout AV canal: **c** spindle-shaped nodal AV cell, **d** star- or spider-shaped nodal AV cell and **e** transitional AV cell

In trout heart, the AV region consists of a layer of compact myocardium which is surrounded by a collagen-rich connective tissue from both sides (Fig. 1). On the atrial side, the AV tissue forms a robust muscle strand that is continuous with the atrial tissue (Fig. 1a). On the opposite side, the AV canal advances as a thin muscle to the ventricle forming a delicate muscle layer around the AV canal (Fig. 1b). The AV tissue supports AV valves which are completely composed of a collagenous connective tissue. The connective tissue extends from the base of the valves as a thin annular layer to the ventricular side and insulates the AV muscle from the lumenal side of the ventricle. Another strand of connective tissue, starting from the boundary zone between the atrium and the ventricle, surrounds the AV muscle from the outside. Owing to these two layers, the AV muscle is laterally surrounded by connective tissue on both sides. Notably, the AV muscle is connected with spongious ventricular trabeculae but isolated from the compact ventricular myocardium by a connective tissue layer.

Based on electrophysiological characteristics, cardiac myocytes of the rainbow trout AV canal were separated in two groups called as “nodal” and “transitional” AV cells, respectively. While the nodal AV myocytes are either star, spider or short spindle shaped (Fig. 1c, d), the transitional AV cells are always spindle shaped (Fig. 1e). Despite a visually smaller size, the nodal cells were significantly larger by electrophysiological measurements than the transitional cells with a capacitive cell size of 30.7 ± 1.87 pF (*n* = 27) and 20.1 ± 0.79 pF (*n* = 35) (*p* < 0.0001), respectively. Moreover, both the nodal and transitional cells usually lacked clear cross striations.

### Membrane potential and inward rectifier K^+^ current

**Fig. 2:**
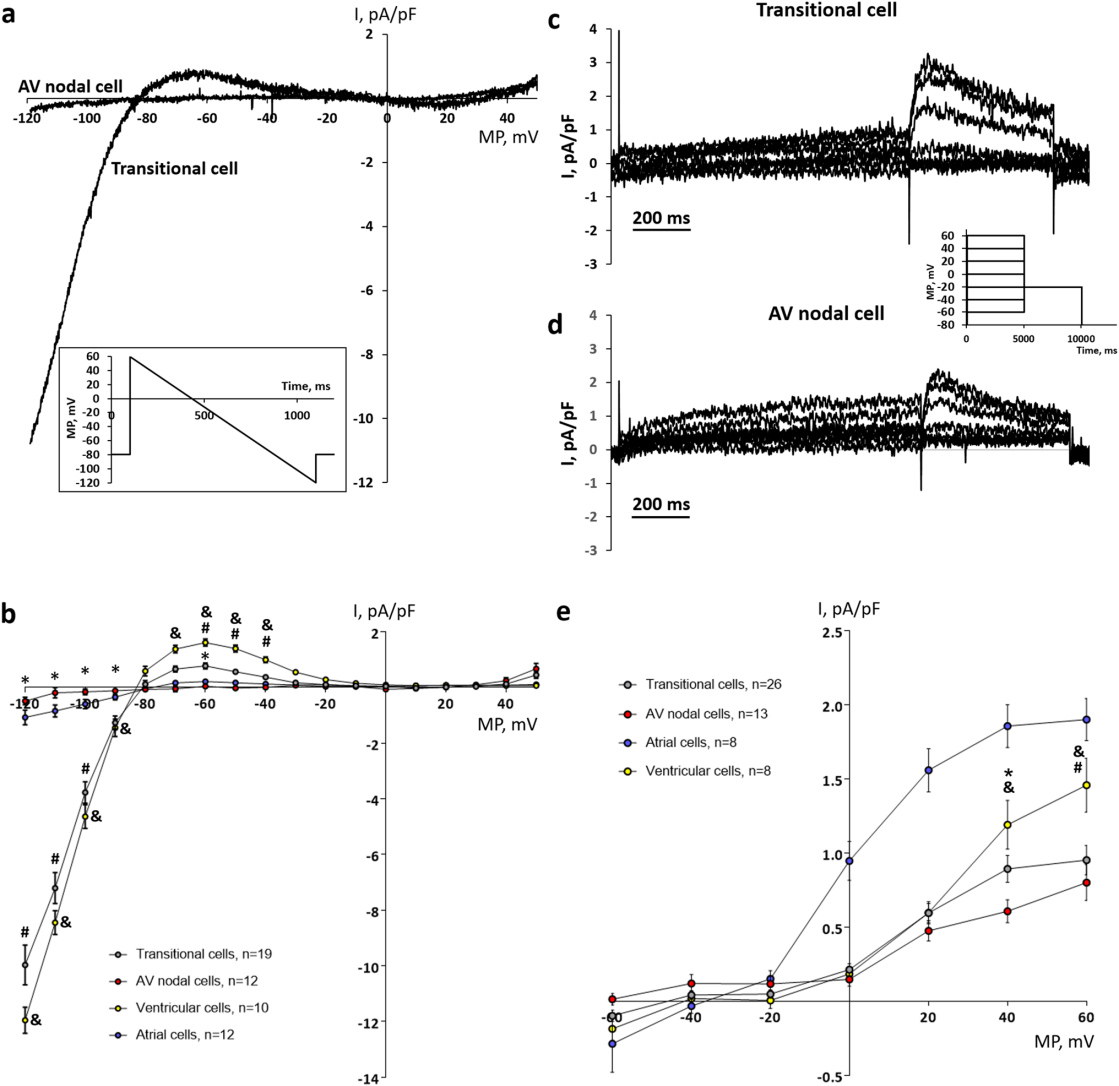
Comparison of the main K^+^ currents in AV canal, ventricle and atrium of the rainbow trout heart. **a** Original tracings of inward rectifier K^+^ current (*I*_K1_) in a representative transitional cell and a typical AV nodal cell. The currents were elicited by a hyperpolarizing ramp (from + 60 to − 120 mV) starting from the holding potential of − 80 mV (inset). **b** *I*–*V* curves of the *I*_K1_ obtained after subtraction of the leakage current in AV nodal cells, transitional cells, atrial myocytes and ventricular myocytes. The results are means ± SEM of current densities from 10 to 19 cells of 6 fish. **c**, **d** Original recordings of delayed rectifier K^+^ current *I*_K_ in a representative transitional cell (**c**) and an AV nodal cell (**d**). The currents were elicited by a double square-pulse depolarization from the holding potential of -80 mV (inset). The density of *I*_Kr_ was measured as a tail current at − 20 mV. (**e**) The mean *I*–*V* curves of the tail *I*_K_ density in AV nodal cells, transitional cells, atrial myocytes and ventricular myocytes. The results are means ± SEM from 8 to 26 cells of 6 fish. In (**b**, **e**) statistically significant differences (*p* < 0.05, two-way ANOVA with Tukey’s multiple comparisons) are indicated between AV nodal and transitional cells (*), between transitional cells and ventricular myocytes (#) and between ventricular myocytes and AV nodal cells (&)

In some myocytes isolated from the AV region, the membrane potential was measured in the current clamp mode. While transitional AV cells had a stable negative membrane potential (*V*_rest_) (− 77.6 ± 1.45 mV, *n* = 18), nodal AV cells seemed to be strongly depolarized (− 17.9 ± 2.2 mV, *n* = 7) and demonstrated irregular fluctuations of membrane potential (not shown). Consistent with these observations, the background inward rectifier K^+^ current (*I*_K1_), which is the main determinant of *V*_rest_, had a very low density (− 0.49 ± 0.14 pA pF^−1^ at − 120 mV) in nodal cells and similar to that of atrial myocytes (− 1.07 ± 0.41 pA pF^−1^ at − 120 mV; *p* > 0.05) (Fig. 2a, b). The depolarized membrane potential of nodal AV cells is partly an artifact due to the imperfect voltage measuring of the whole-cell current clamp in myocytes with low I_K1_ density: ion leakage between cell membrane and pipette partly offsets the repolarizing effect of I_K1_. In contrast to the nodal cells, the transitional cells demonstrated a large I_K1_. However, the inward and outward components of the I_K1_ in the transitional cells were not quite as large as in the ventricular cells (*p* < 0.05) (Fig. 2b). Thus, nodal cells could be electrophysiologically distinguished from transitional cells based on their small I_K1_ and less negative *V*_rest_.

Noteworthy, hyperpolarization of both types of AV cells to − 80 to − 160 mV from the holding potential of − 35 mV did not induce any inward time-dependent current which would be considered as the pacemaker current (*I*_f_).

### A delayed rectifier K^+^ current

In rainbow trout atrial and ventricular myocytes, the delayed rectifier K^+^ current mainly consists of the rapid component, *I*_Kr_ (Hassinen et al. 2008). However, the presence of a small slow delayed rectifier, *I*_Ks_, cannot be excluded, since it has been found in zebrafish and crucian carp myocytes (Hassinen et al. 2011; Abramochkin et al. 2018). Therefore, we use the term delayed rectifier (I_K_) for the time-dependent K^+^ outward current induced by depolarization. In both transitional and nodal AV cells, depolarization induced an outward I_K_ with a prominent tail current (Fig. 2c, d). The density of I_K_ was similar in transitional and nodal cells, but at + 40 mV, the current was slightly larger in transitional than nodal cells (*p* < 0.05). In both types of AV myocytes, I_K_ was smaller than in ventricular myocytes and much smaller than in atrial myocytes (*p* < 0.001) (Fig. 2e).

### Na^+^ and Ca^2+^ currents

**Fig. 3:**
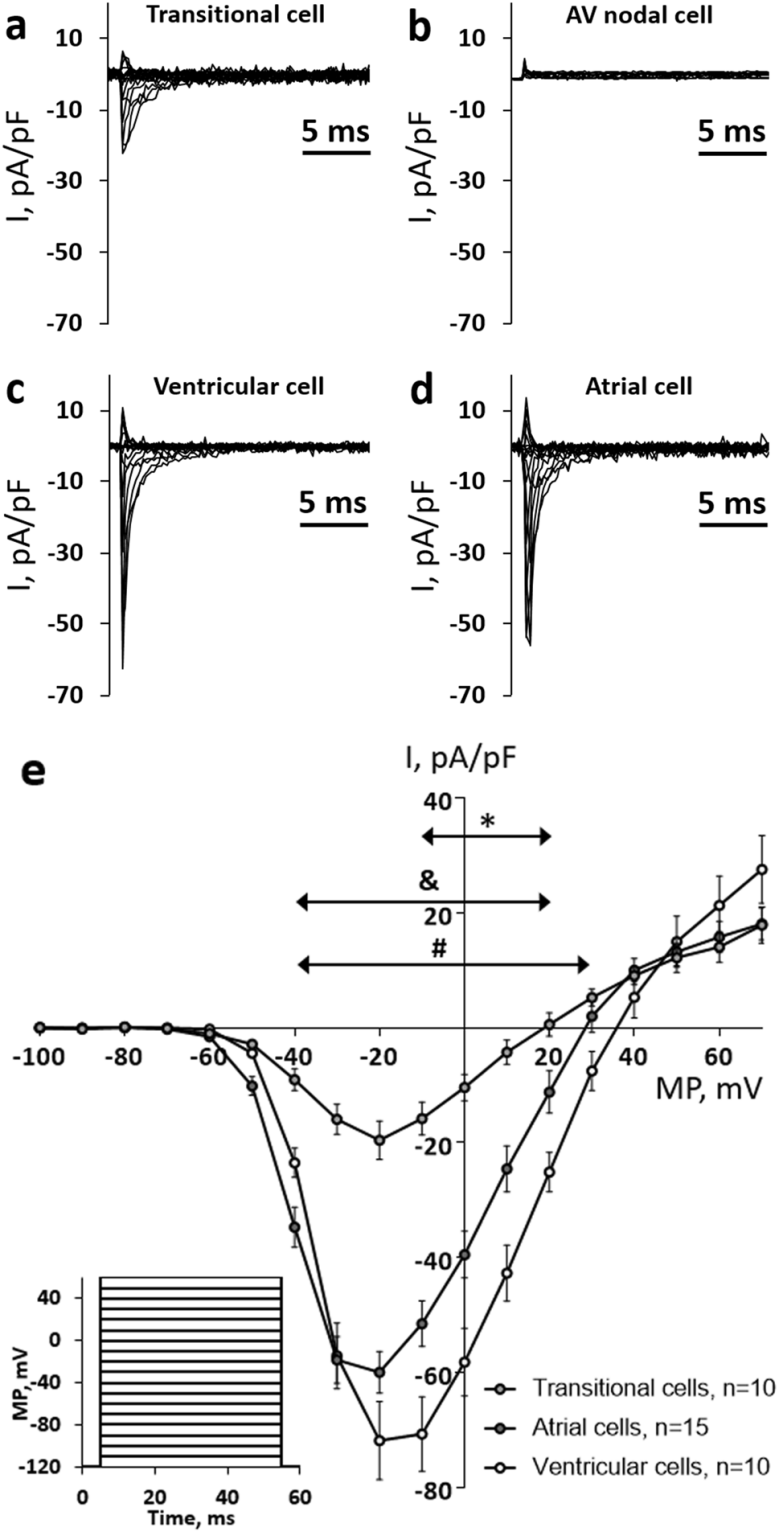
Comparison of the fast sodium current (*I*_Na_) of AV canal, ventricle and atrium of the rainbow trout heart. **a**–**d** Original recordings of *I*_Na_ in a representative transitional cell (**a**), a AV nodal cell (**b**), a working ventricular myocyte (**c**) and a working atrial myocyte (**d**). I_Na_ was elicited by depolarization square-pulses from the holding potential of − 120 mV (inset). (**e)** *I*–*V* curves of I_Na_ recorded in transitional cells, atrial myocytes and ventricular myocytes. The results are means ± SEM of 10–15 cells from 5 fish. Statistically significant differences (*p* < 0.05, two-way ANOVA with Tukey’s multiple comparisons) are indicated between atrial and ventricular myocytes (*), between transitional cells and atrial myocytes (&), between transitional cells and ventricular myocytes (#)

All studied transitional AV cells had a distinct TTX-sensitive Na^+^ current, *I*_Na_ (Fig. 3a), albeit it was smaller than *I*_Na_ of atrial and ventricular myocytes (Fig. 3e). In transitional cells, I_Na_ had a typical current–voltage (*I*–*V*) relationship of I_Na_ with a peak current at − 20 mV. Unlike the transitional cells, the nodal myocytes completely lacked I_Na_.

**Fig. 4:**
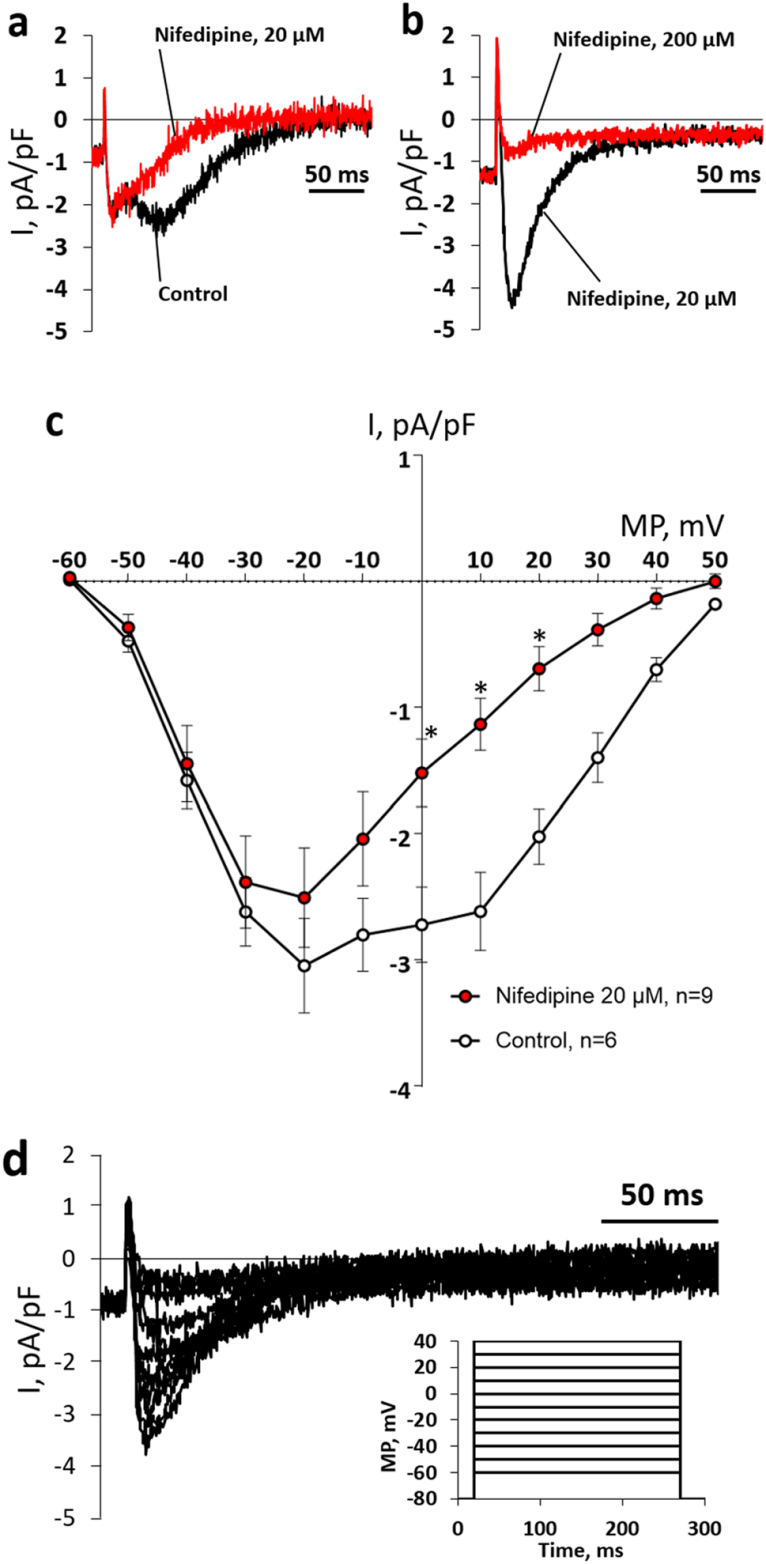
Calcium currents (*I*_Ca_) in nodal myocytes of rainbow trout AV canal. **a**, **b** Original recordings of *I*_Ca_ elicited by square-pulse depolarizations from the holding potential of − 80 mV to − 10 mV in two representative nodal myocytes. **a** Under control conditions, the current tracing has two inward current peaks, an early and a late one. Nifedipine (20 µM) abolishes the late current peak. **b** A high concentration of nifedipine (200 µM) almost eliminates the early peak of *I*_Ca_ which was resistant to 20 µM nifedipine. **c** *I*–*V* curves of *I*_Ca_ under control conditions and in the presence of 20 µM nifedipine. The results are means ± SEM of 6 and 9 cells from 5 fish for control and nifedipine experiments, respectively. The currents were elicited by square wave pulses from the holding potential of − 80 mV (inset). An asterisk (*) indicates a significant effect of nifedipine (*p* < 0.05, two-way ANOVA with Tukey’s multiple comparisons test). **d** An original current tracing of 20 µM nifedipine-resistant component of *I*_Ca_ in a representative nodal AV myocyte

In addition to the TTX-sensitive I_Na_, a relatively fast TTX-resistant (0.3 µM) inward current could be observed in nodal AV cells during depolarization to membrane voltages more positive than − 50 mV (Fig. 4). When 200 µM nifedipine (a blocker of Ca^2+^ currents) was included in the external saline, this current was eliminated. Thus, this kinetically fast inward current is a Ca^2+^ current (I_Ca_) (Fig. 4b). Furthermore, the *I*–*V* curve of the nodal *I*_Ca_ was characterized by two inward peaks: the first at about − 20 mV and the second at about + 10 mV (Fig. 4c). Application of 10 µM nifedipine, the standard concentration used for inhibiting of the L-type Ca^2+^ current (*I*_CaL_), did not completely abolish the nodal I_Ca_. However, at a slightly higher concentration (20 µM), nifedipine abolished the late peak at + 10 mV while the early peak remained almost untouched (Fig. 4a). The early peak could be almost completely prevented with 10 times higher concentration of nifedipine (200 µM) (Fig. 4b). Thus, in nodal cells, I_Ca_ consists of two components. The larger component is insensitive to 10 µM nifedipine, has the peak current at − 20 mV (Fig. 4c), and therefore, is not I_CaL_. In transitional AV cells, no inward current was observed in the presence of 0.3 µM TTX and 20 µM nifedipine (data not shown).

### Transcript expression

Transcript expression of 42 genes encoding Ca^2+^, K^+^ and Na^+^ channels and the islet-1 transcription factor were studied by real-time quantitative PCR (qPCR). mRNA levels were normalized to the expression of DnaJ (hsp40) subfamily A member 2 (dnaja2) whose transcript expression in different cardiac compartments is more stable than the expression of commonly used reference genes such as β-actin, glyceraldehyde phosphate dehydrogenase and ribosomal proteins (Vornanen et al. 2005; Hassinen et al. 2015). Since it was not possible to separate the AV muscle cleanly from the working myocardia, the narrow AV tissue ring was cut in two parts. AV-atr and AV-ven pieces are thought to include some atrial or ventricular myocardium in addition to the AV tissue, meaning that the mRNA expressions measured from them are likely to represent a mixture of expression profiles of actual AV tissue and working myocardium.

### Islet-1

**Fig. 5:**
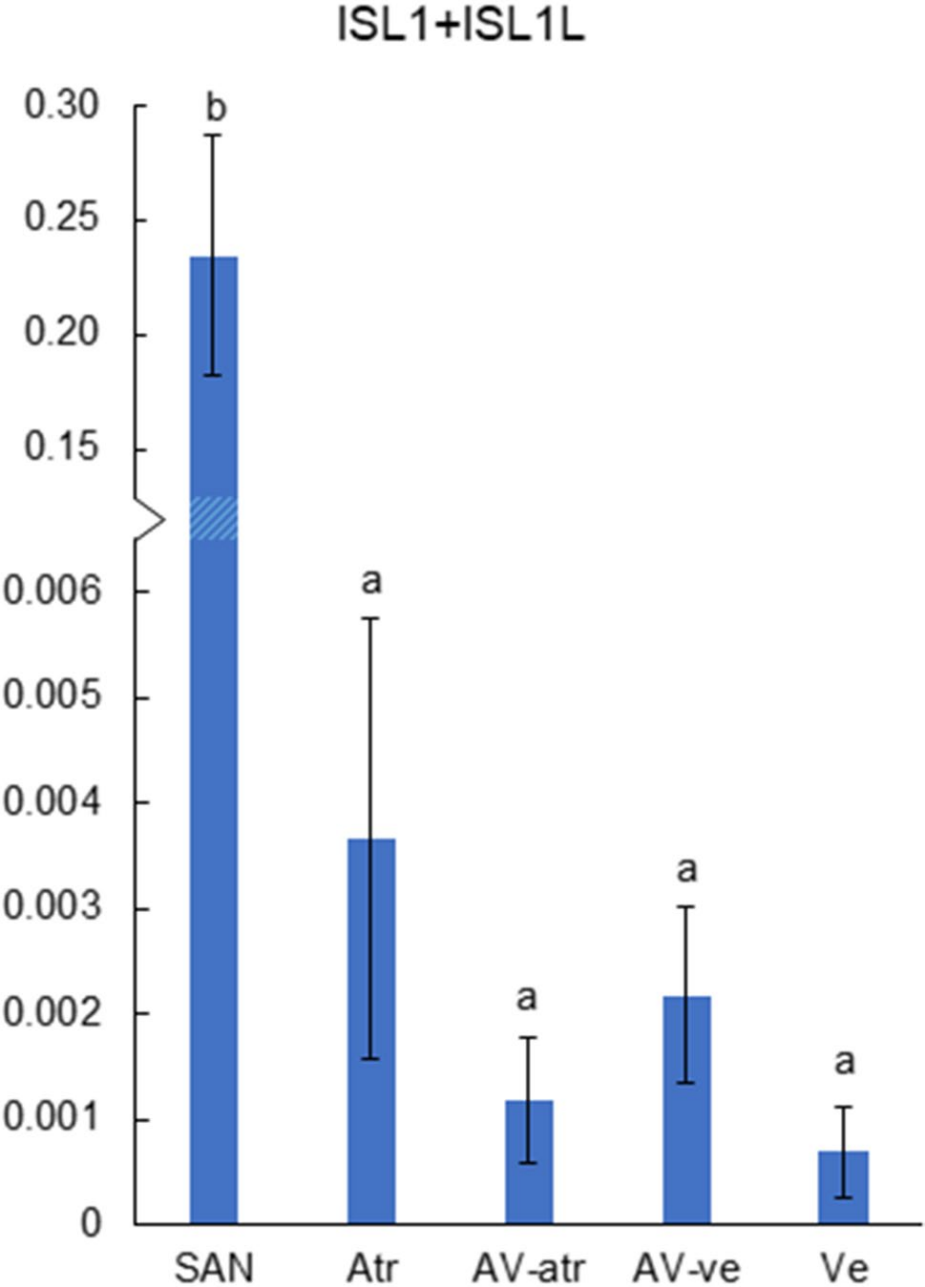
The total transcript expression of Islet isoforms (ISL1 + ISL1L), transcription factor genes, in different parts of rainbow trout heart. Transcript abundances were normalized to the expression of the reference gene DnaJA2 (*n* = 5, mean ± S.E.M.). Note the break in *y*-axis. Statistically significant differences in gene expression between cardiac compartments are indicated by dissimilar letters (*p* < 0.05)

Islet-1 is a marker of pacemaker cells of the fish sinoatrial tissue (Tessadori et al. 2012; Stoyek et al. 2016). Consistent with this the expression of islet-1 was significantly higher in SAN than in any other part of the trout heart (*p* < 0.05) (Fig. 5, Table 2). However, mRNA expression of islet-1 was only 3–4% of the expression level of KCNH and SCN channels suggesting that pacemaker cells make up only a minor fraction of the SAN tissue. The expression of Islet-1 in the AV tissue did not differ from those of atrium and ventricle (*p* > 0.05); the expression level of Islet-1 outside SAN was 1.5% or less.

**Table 2:** Fold changes of gene expression between different cardiac compartments

	Fold change AV-atr/atr	Fold change AV-ven/ven	Fold change Atr/ven	Fold change SV/atr	Fold change SV/ven	Fold change SV/AV-atr	Fold change SV/AV-ven
SCN4Aba	0.43 ± 0.10 ↓	0.34 ± 0.04 ↓	0.80 ± 0.13	0.86 ± 0.16	0.69 ± 0.13	1.99 ± 0.36	2.04 ± 0.37
SCN4Abb	0.62 ± 0.11	2.80 ± 0.55 ↑	8.71 ± 1.46 ↑	7.86 ± 1.52 ↑	68.5 ± 13.2 ↑	12.70 ± 2.46 ↑	24.45 ± 4.73 ↑
SCN5LAba	0.57 ± 0.08	0.31 ± 0.08 ↓	3.06 ± 0.49 ↑	0.74 ± 0.15	2.27 ± 0.45	1.30 ± 0.26	7.37 ± 1.46 ↑
SCN5LAbb	0.74 ± 0.12	0.86 ± 0.09	2.33 ± 0.45 ↑	1.15 ± 0.23	2.68 ± 0.55 ↑	1.56 ± 0.32	3.12 ± 0.63 ↑
SCN tot	0.57 ± 0.06	0.58 ± 0.07	2.13 ± 0.27 ↑	3.13 ± 0.59 ↑	6.66 ± 1.25 ↑	5.45 ± 1.02 ↑	11.56 ± 2.17 ↑
Kir2.1a	0.58 ± 0.07	1.17 ± 0.18	0.66 ± 0.09	1.49 ± 0.19	0.98 ± 0.12	2.55 ± 0.32 ↑	0.84 ± 0.11
Kir2.1b	1.14 ± 0.36	1.41 ± 0.34	0.70 ± 0.22	2.98 ± 0.85	2.09 ± 0.59	2.61 ± 0.74	1.48 ± 0.42
Kir2.2a	1.26 ± 0.21	0.32 ± 0.03	0.37 ± 0.02	4.34 ± 1.17	1.60 ± 0.43	3.45 ± 0.93	4.97 ± 1.34
Kir2.2b	0.68 ± 0.38	0.38 ± 0.18	1.42 ± 0.87	2.49 ± 0.45	3.54 ± 0.63 ↑	3.67 ± 0.66 ↑	9.42 ± 1.69 ↑
Kir2.4	0.29 ± 0.23	0.55 ± 0.07 ↓	0.0010 ± 0.0007 ↓	1.85 ± 0.48	0.00 ± 0.00 ↓	6.38 ± 1.66	0.004 ± 0.001 ↓
Kir2 tot	0.71 ± 0.22	0.55 ± 0.07 ↓	0.03 ± 0.01 ↓	2.37 ± 0.32	0.07 ± 0.01 ↓	3.32 ± 0.45 ↑	0.14 ± 0.02 ↓
KCNH2aa	1.05 ± 0.31	3.60 ± 0.81	1.89 ± 0.46	6.31 ± 1.30 ↑	11.94 ± 2.47 ↑	6.00 ± 1.24 ↑	3.32 ± 0.69 ↑
KCNH2ab	1.68 ± 0.73	1.16 ± 0.29	0.76 ± 0.31	1.41 ± 0.45	1.06 ± 0.34	0.84 ± 0.27	0.92 ± 0.29
KCNH2ba	0.93 ± 0.15	2.13 ± 0.41	4.01 ± 0.51	2.33 ± 0.36 ↑	9.35 ± 1.46 ↑	2.50 ± 0.39 ↑	4.39 ± 0.69 ↑
KCNH2bb	0.76 ± 0.23	1.13 ± 0.21	3.38 ± 0.79	2.43 ± 0.35 ↑	8.21 ± 1.19 ↑	3.18 ± 0.46 ↑	7.25 ± 1.05 ↑
KCNH6a	0.94 ± 0.10	0.62 ± 0.08	1.78 ± 0.25	0.76 ± 0.12	1.34 ± 0.21	0.81 ± 0.13	2.16 ± 0.35
KCNH6b	1.29 ± 0.34	1.29 ± 0.18	0.96 ± 0.29	0.58 ± 0.18	0.56 ± 0.17	0.45 ± 0.14	0.43 ± 0.14
KCNH7aa	0.91 ± 0.31	4.68 ± 1.41	470.1 ± 119.5 ↑	0.45 ± 0.09	212.3 ± 44.2 ↑	0.50 ± 0.10	45.35 ± 9.43 ↑
KCNH7ab	0.91 ± 0.05	48.7 ± 19.9	1584.3 ± 247.4 ↑	0.97 ± 0.16	1541.4 ± 249.4 ↑	1.06 ± 0.17	31.65 ± 5.12 ↑
KCNH7ba	0.73 ± 0.09	3.22 ± 0.68 ↑	83.85 ± 17.84 ↑	0.36 ± 0.06	29.98 ± 5.03 ↑	0.49 ± 0.08 ↓	9.31 ± 1.56 ↑
KCNH7bb	1.02 ± 0.25	2.52 ± 0.66	2.73 ± 0.90	4.76 ± 0.18 ↑	13.00 ± 0.49 ↑	4.66 ± 0.18 ↑	5.16 ± 0.19 ↑
KCNH tot	0.93 ± 0.08	0.63 ± 0.09	2.19 ± 0.29 ↑	0.77 ± 0.12	1.69 ± 0.26	0.83 ± 0.13	2.67 ± 0.41 ↑
HCN1a	1.29 ± 0.64	4.34 ± 1.23	0.64 ± 0.26	63.9 ± 9.32 ↑	41.08 ± 5.99 ↑	49.49 ± 7.21 ↑	9.47 ± 1.38 ↑
HCN1b	1.01 ± 0.15	2.43 ± 0.55	11.12 ± 1.28 ↑	1.09 ± 0.23	12.15 ± 2.60 ↑	1.09 ± 0.23	4.99 ± 1.07 ↑
HCN2aa	2.03 ± 0.28	0.35 ± 0.07 ↓	0.41 ± 0.08 ↓	4.47 ± 0.82 ↑	1.82 ± 0.33	2.21 ± 0.40	5.24 ± 0.96 ↑
HCN2ab	1.91 ± 0.48	1.80 ± 0.26	2.07 ± 0.53	16.6 ± 1.39 ↑	34.22 ± 2.86 ↑	8.67 ± 0.73 ↑	19.0 ± 1.59 ↑
HCN2ba	0.47 ± 0.14	4.71 ± 1.21 ↑	1.23 ± 0.57	36.1 ± 4.40 ↑	44.27 ± 5.40 ↑	77.22 ± 9.41 ↑	9.40 ± 1.15 ↑
HCN2bb	1.16 ± 0.12	0.63 ± 0.05 ↓	1.00 ± 0.09	4.16 ± 0.22 ↑	4.17 ± 0.22 ↑	3.58 ± 0.19 ↑	6.63 ± 0.35 ↑
HCN3a	4.29 ± 0.96 ↑	25.3 ± 5.04 ↑	7.14 ± 1.45 ↑	59.9 ± 19.58 ↑	427.5 ± 139.9 ↑	13.96 ± 4.57 ↑	16.92 ± 5.53 ↑
HCN3b	9.78 ± 7.10	2.01 ± 0.75	0.98 ± 0.44	64.0 ± 4.80 ↑	78.60 ± 5.90 ↑	6.54 ± 0.49 ↑	31.22 ± 2.34 ↑
HCN4a	1.06 ± 0.11	6.80 ± 1.26 ↑	18.40 ± 1.59 ↑	3.49 ± 0.66 ↑	64.19 ± 12.09 ↑	3.29 ± 0.62 ↑	9.44 ± 1.78 ↑
HCN4ba	0.63 ± 0.14	2.59 ± 0.80	1.27 ± 0.43	9.06 ± 2.00 ↑	11.55 ± 2.55 ↑	14.32 ± 3.16 ↑	4.46 ± 0.99 ↑
HCN4bb	0.64 ± 0.21	0.96 ± 0.18	0.92 ± 0.47	19.9 ± 1.86 ↑	18.36 ± 1.72 ↑	24.96 ± 2.34 ↑	19.17 ± 1.79 ↑
HCN tot	1.09 ± 0.12	3.73 ± 0.56 ↑	6.87 ± 0.64 ↑	5.07 ± 0.84 ↑	34.85 ± 5.79 ↑	4.65 ± 0.77 ↑	9.34 ± 1.55 ↑
CACNA1C	0.85 ± 0.13	0.99 ± 0.30	0.63 ± 0.14	1.65 ± 0.38	1.04 ± 0.24	1.94 ± 0.45	1.05 ± 0.24
CACNA1Daa	1.18 ± 0.29	2.22 ± 0.24	0.62 ± 0.17	10.7 ± 1.28 ↑	6.63 ± 0.79 ↑	9.03 ± 1.08 ↑	2.99 ± 0.36 ↑
CACNA1Dab	2.20 ± 0.70	0.88 ± 0.27	0.39 ± 0.13	5.45 ± 0.51 ↑	2.15 ± 0.20	2.47 ± 0.23	2.44 ± 0.22
CACNA1Dba	0.82 ± 0.22	2.39 ± 0.70	1.18 ± 0.18	77.3 ± 6.44 ↑	91.56 ± 7.63 ↑	94.76 ± 7.90 ↑	38.35 ± 3.20 ↑
CACNA1Dbb	1.35 ± 0.20	1.61 ± 0.30	1.11 ± 0.13	2.62 ± 0.29 ↑	2.91 ± 0.32 ↑	1.94 ± 0.21	1.81 ± 0.20
CACNA1Ga	3.25 ± 0.95 ↑	4.04 ± 1.25 ↑	0.33 ± 0.12	7.35 ± 1.16 ↑	2.44 ± 0.39	2.26 ± 0.36	0.60 ± 0.10
CACNA1Gb	1.03 ± 0.58	2.87 ± 0.61	1.50 ± 0.66	11.8 ± 2.14 ↑	17.65 ± 3.22 ↑	9.11 ± 1.66 ↑	6.15 ± 1.12 ↑
CACNA1Ha	1.61 ± 0.48	2.06 ± 0.29	0.97 ± 0.22	4.19 ± 0.27 ↑	4.08 ± 0.26 ↑	2.61 ± 0.17 ↑	1.97 ± 0.13
CACNA1Hb	1.02 ± 0.22	0.87 ± 0.20	0.57 ± 0.10	2.02 ± 0.17	1.14 ± 0.09	1.99 ± 0.17	1.31 ± 0.11
CACNA1Ia	1.32 ± 0.72	2.09 ± 0.88	1.15 ± 0.50	2.47 ± 0.18	2.84 ± 0.21	1.86 ± 0.14	1.36 ± 0.10
CACNA1Ib	1.38 ± 0.76	2.47 ± 0.97	1.18 ± 0.74	0.32 ± 0.12	0.38 ± 0.14	0.23 ± 0.09	0.15 ± 0.06
CACNA1 tot	1.13 ± 0.17	1.53 ± 0.39	0.59 ± 0.12	2.67 ± 0.38 ↑	1.57 ± 0.22	2.38 ± 0.33 ↑	1.03 ± 0.14
ISL1 + ISL1L	0.33 ± 0.16	3.14 ± 1.19	5.28 ± 3.03	64.0 ± 14.37 ↑	338.0 ± 75.9 ↑	196.2 ± 44.0 ↑	107.5 ± 24.1 ↑

Values are mean ± SEM

(↑) indicates statistically significant upregulation and (↓) indicates significant downregulation of the mRNA level

### Inward rectifier K^+^ channels

**Fig. 6:**
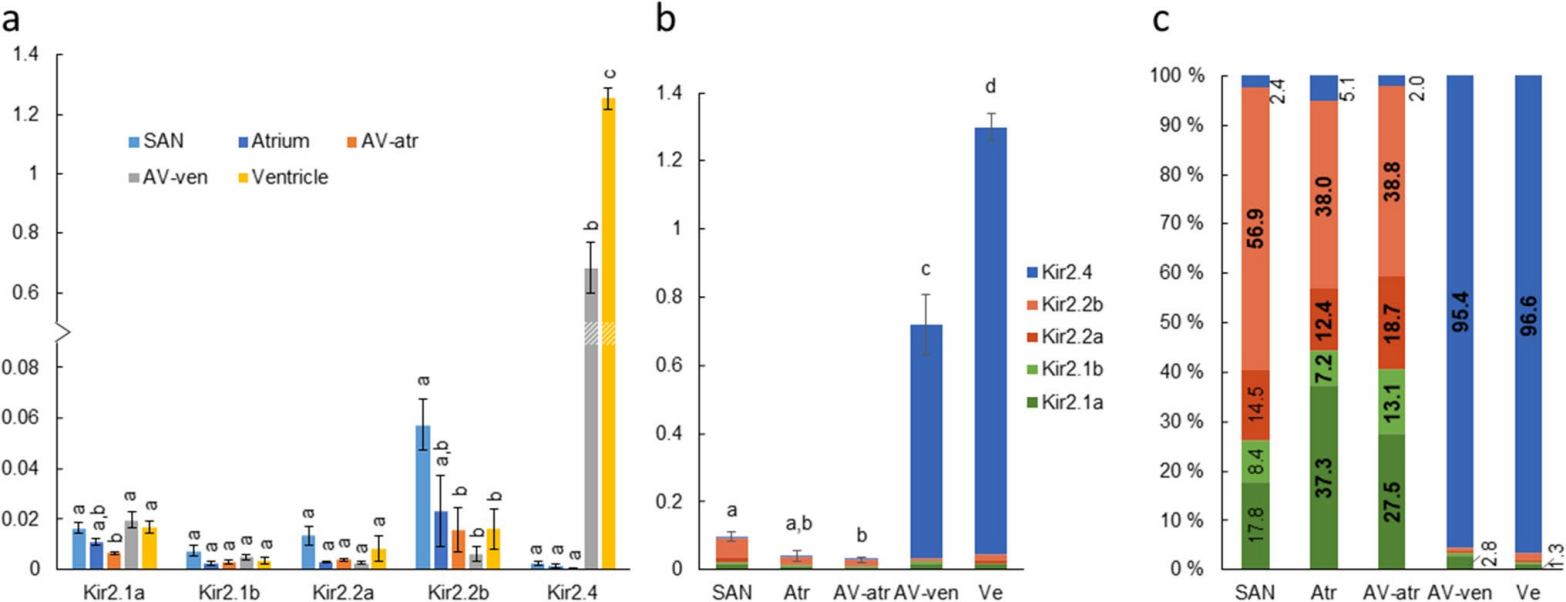
Transcript abundance of Kir2.1, -2.2 and -2.4 isoforms in cardiac compartments. Since Kir2.1aa and -ab share high sequence similarity, their mRNAs were measured by primers identical for both isoforms. Transcripts of Kir2.2aa and -ab as well as Kir2.2ba and -bb were measured similarly. **a** Transcript abundances of Kir2 genes normalized to the expression of reference gene DnaJA2 (*n* = 5, mean ± S.E.M.). Statistically significant differences in the expression of each gene between cardiac compartments are indicated by dissimilar letters (*p* < 0.05). **b** Stacked bars represent the total Kir2 transcript abundance (mean ± S.E.M.) in different cardiac compartments. **c** Percentage of each Kir2 isoform from total Kir2 transcript abundance. The percentage value of the most expressed isoform is represented as bold (if two or more isoforms are statistically equally expressed, both/all of them are marked as bold)

The total Kir2 mRNA amount was highest in ventricle (*p* < 0.05) being 2, 13, 32 and 44 times as high as in AV-ven, SAN, atrium and AV-atr, respectively (Fig. 6b). Notably, the total Kir2 mRNA abundance was lower in AV-ven than in ventricle, while it was similar in AV-atr and atrium (Table 2). The isoform composition of Kir2 channels was markedly different between different cardiac compartments. In ventricle and AV-ven, Kir2.4 represented 96.6 ± 0.9 and 95.4 ± 0.6% of all Kir2 transcripts, respectively, while Kir2.4 was weakly expressed (2.0–5.1%) in other cardiac compartments (Fig. 6c). Indeed, the amount of Kir2.4 mRNA in the ventricle was approximately 2-, 520-, 960- and 3300-fold higher (*p* < 0.05) than in AV-ven, SAN, atrium and AV-atr, respectively (Fig. 6a). In SAN, atrium and AV-atr Kir2 isoform composition was more variable, but different Kir2.2 paralogs formed most (50.4–71.4%) of the Kir2 channels in these tissues. The AV tissue is clearly a transition zone (caudal to cranial direction) in which Kir2 expression changes from very low to high and mainly from Kir2 to mainly Kir4 type.

### Delayed rectifier K^+^ channels

**Fig. 7:**
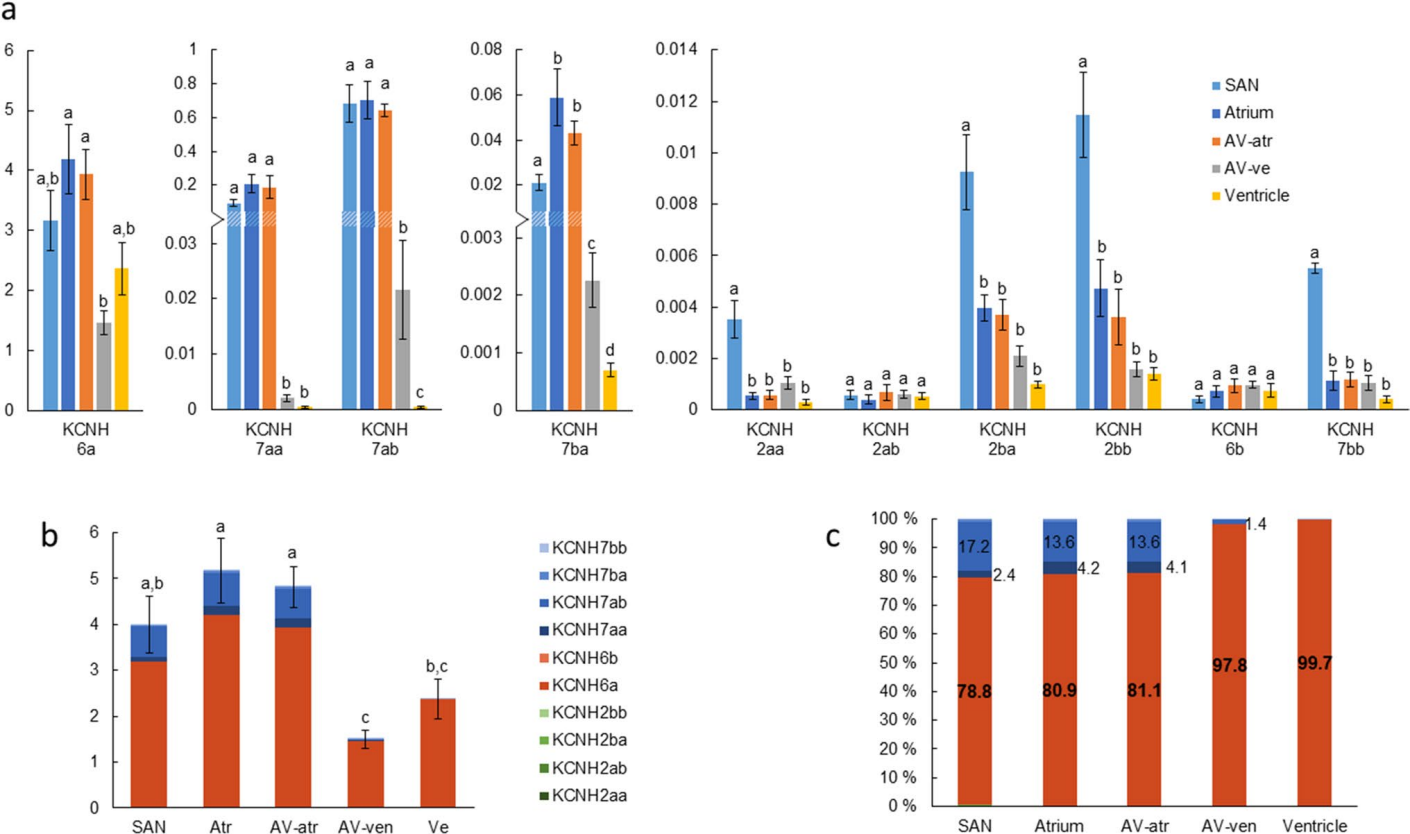
Transcript expression of KCNH2, -6 and -7 isoforms encoding delayed rectifier K^+^ channels. **a** Transcript expression level of different KCNH genes is highly different, thus they are represented in diagrams with different scales. KCNH6a is the most expressed isoform in all cardiac compartments. **b** Total expression of KCNH2, -6 and -7 isoforms (mean ± S.E.M.). **c** Relative proportion (%) of each KCNH isoform from all KCNH transcript expression shows that KCNH6a is the main isoform whereas KCNH2 isoforms are negligible in all cardiac chambers, and relative proportion of KCNH7ab is higher in SAN, AV-atr and atrium than in AV-ven and ventricle

Expression of all known delayed rectifier K^+^ channels encoding KCNH2, -6 and -7 isoforms in rainbow trout were studied (Fig. 7). The total KCNH mRNA level was higher in atrium and AV-atr than in AV-ven and ventricle (*p* < 0.05) (Fig. 7b). KCNH expression in SAN was equal to that of atrium, AV-atr and ventricle but higher than in AV-ven. KCNH6a was the most highly expressed KCNH isoform in all cardiac compartments representing 78.8–99.7% of all KCNH transcripts (Fig. 7c). Its expression was lower in AV-ven than in atrium and AV-atr (Fig. 7a). KCNH7 paralogs (except to KCNH7bb) were more abundant in SAN, AV-atr and atrium than in AV-ven and ventricle (*p* < 0.05). KCNH7ab represented 13.6–17.2% of all KCNH transcripts in atrium, AV-atr and SAN whereas its percentual value was negligible in AV-ven and ventricle. Expression of KCNH2 paralogs (orthologs to the human erg) was very low in all cardiac compartments of the trout heart. In respect to KCNH channels, the AV tissue is a transitional zone (caudal to cranial direction) from high to low expression level and from the mixed KCNH6/KCNH7 type to more homogenous KCNH6 type.

### Na^+^ channels

**Fig. 8:**
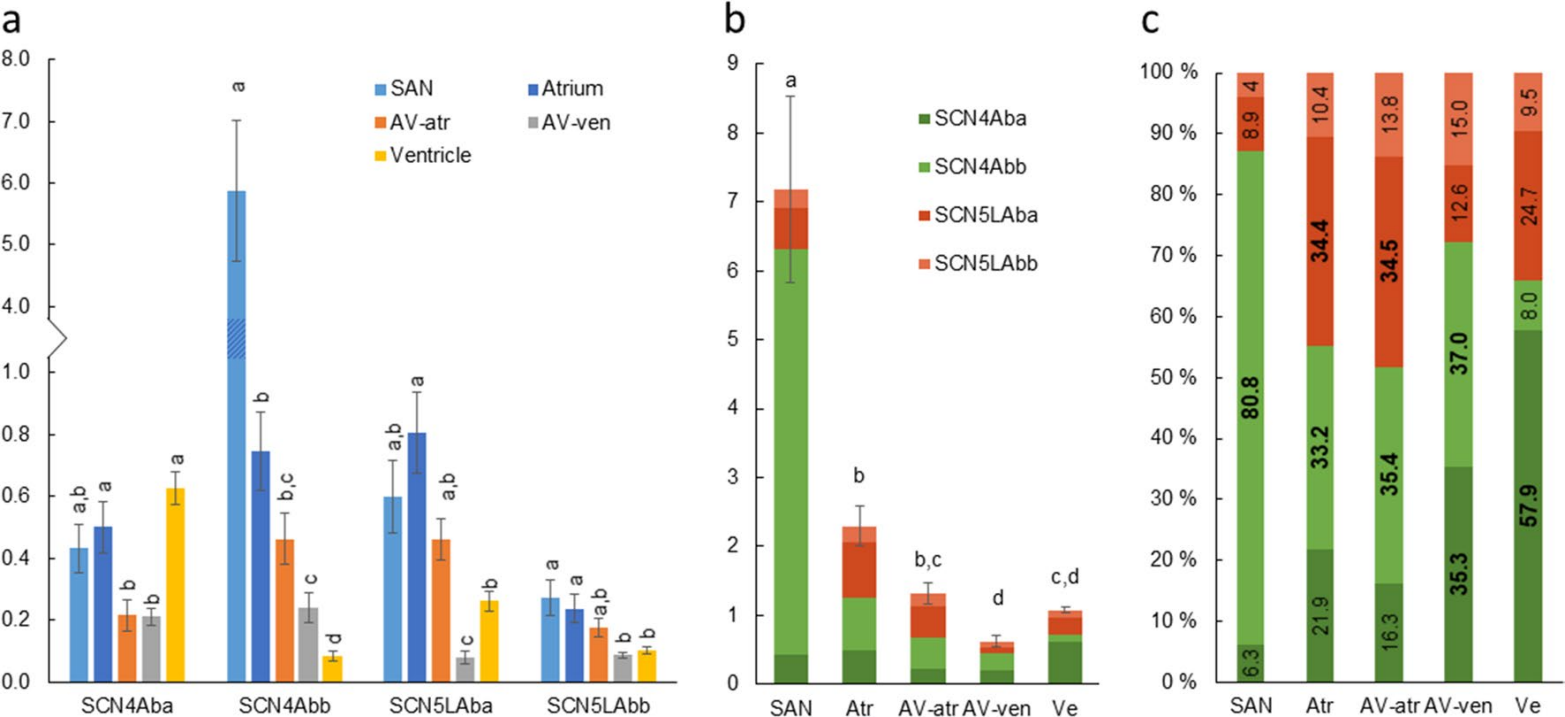
Transcript expression of SCN genes encoding Nav1.4 and Nav1.5 channels. **a** Transcript abundances of SCN genes normalized to the expression of reference gene DnaJA2 (*n* = 5, mean ± S.E.M.). Statistically significant differences in the expression of each gene between cardiac compartments are indicated by dissimilar letters (*p* < 0.05). **b** Total expression of SCN4A and -5LA isoforms in each cardiac compartment (mean ± S.E.M.) shows that SCN expression in SAN is higher than in other cardiac parts. **c** Relative proportion of each SCN isoform in cardiac compartments. SCN4Aba is more abundant in SAN and less abundant in ventricle than in other cardiac parts. The percentage value of the most expressed isoform is represented as bold (if two or more isoforms are statistically equally expressed, both/all of them are marked as bold)

Expression of three Nav1.4 and three Nav1.5 encoding SCN paralogs (SCN4A/5LAa, -ba and -bb) were studied from atrium and ventricle (Fig. 8). Since the expressions of SCN4Aa and SCN5LAa were very low (0.003–0.03% of all SCN transcripts) in these tissues, the study of other tissues was limited to the four most expressed Na^+^ channel isoforms. Surprisingly, the total expression of SCN transcripts among all cardiac compartments was highest in the SAN (*p* < 0.05) (Fig. 8b). The total SCN mRNA amount was significantly higher in atrium than in ventricle, but even if the expression seemed to be lower in the AV area than in the atrial myocardium, the difference was not statistically significant either for AV-atr or AV-ven (Fig. 8b). However, if the data from AV-atr and AV-ven are combined, then atrial expression exceeds AV expression (*p* < 0.05). Although SCN4 paralogs formed most of the Na^+^ channels transcripts in all cardiac compartments, there was some variation in the isoform composition between tissues (Fig. 8c). The relative amount of SCN4Abb was highest in SAN (80.8 ± 2.9%) and lowest in ventricle (8.0 ± 1.5%) (Fig. 8a). In atrium and AV-atr, SCN4Abb and -5LAba were the most expressed isoforms. In ventricle, SCN4Aba was the dominating isoform (57.9 ± 3.3%), whereas SCN4Aba (35.3 ± 4.0%) and SCN4Abb (37.0 ± 4.7%) together dominated in AV-ven. In summary, the expression level of Na^+^ channels appears to reach a minimum in the AV tissue (AV-ven).

### Ca^2+^ channels

**Fig. 9:**
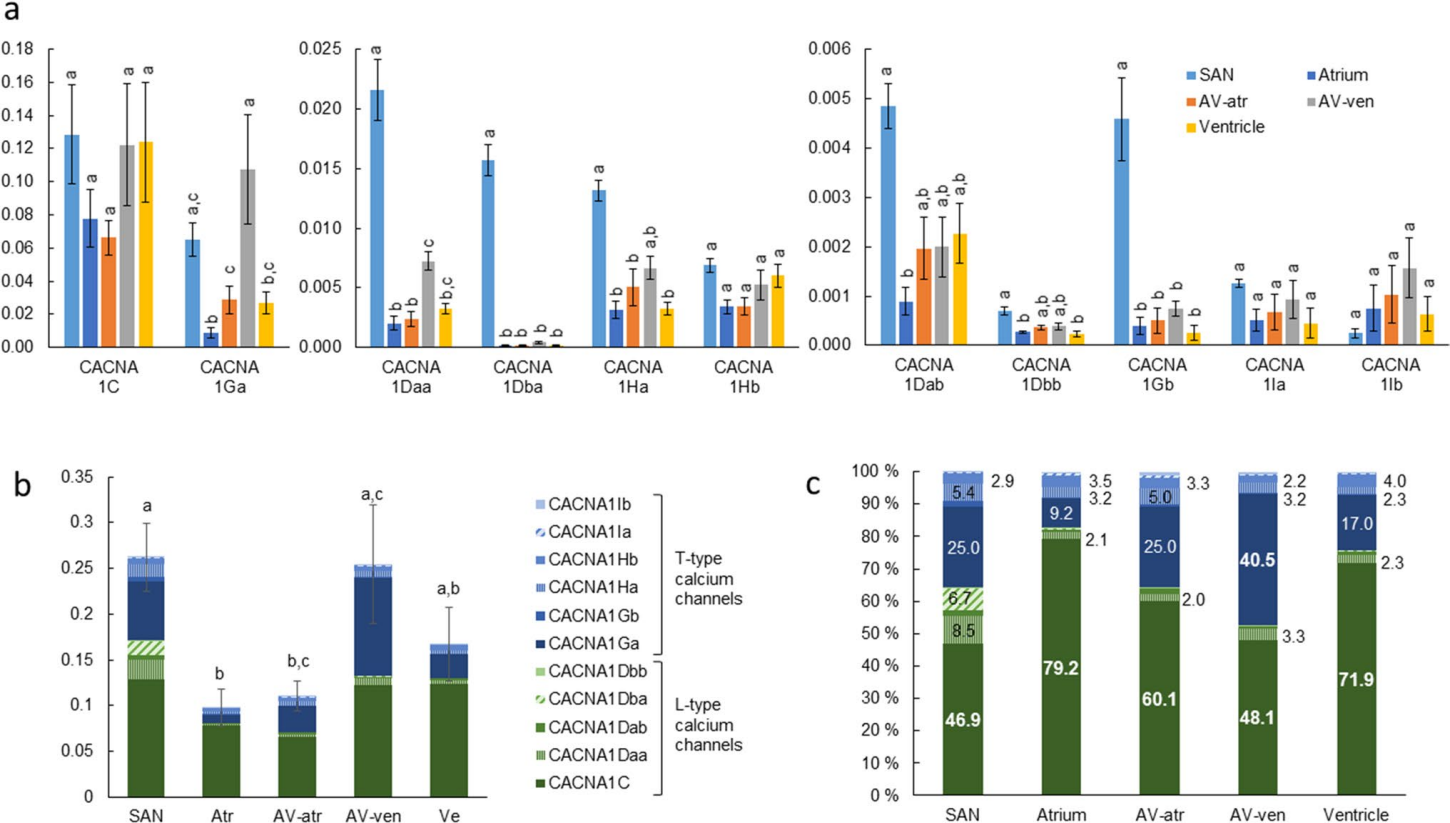
Transcript expression of L- and T-type Ca^2+^ channel encoding genes. **a** Transcript abundance of different CACNA1 genes varies thus they are represented in diagrams with different y-axis scales. **b** Expression of CACNA1 genes are represented as stacked bars to represent their total expression in each cardiac compartment. **c** Relative proportion (%) of each CACNA1 gene

Transcript expression of L- (CACNA1C and -D) and T-type (CACNA1G, -H and -I) Ca^2+^ channels was studied from all five compartments of the trout heart (Fig. 9). The total amount of Ca^2+^ channel transcripts was clearly higher in SAN and AV-ven than other cardiac tissues with the exception of the ventricle (*p* < 0.05) (Fig. 9b). No differences existed in the total Ca^2+^ channel transcripts between atrium, AV-atr and ventricle. Although CACNA1C (Cav1.2) and CACNA1Ga (Cav3.1a) were the most expressed isoforms in all cardiac tissues, there were some notable differences in isoform composition between cardiac compartments (Fig. 9a). The total amount of T-type Ca^2+^ channel mRNAs was higher in AV-ven than in ventricle, atrium and AV-atr (*p* < 0.05). CACNA1Ga transcripts were more abundant in the AV-atr and AV-ven than in the corresponding working myocardia. Notably, expression levels of CACNA1Daa, -Dba, -Ha and -Gb were significantly higher in SAN than in all other cardiac compartments. In brief, the AV tissue is characterized by abundant expression of the kinetically fast T-type Ca^2+^ channels.

### Pacemaker channels

**Fig. 10:**
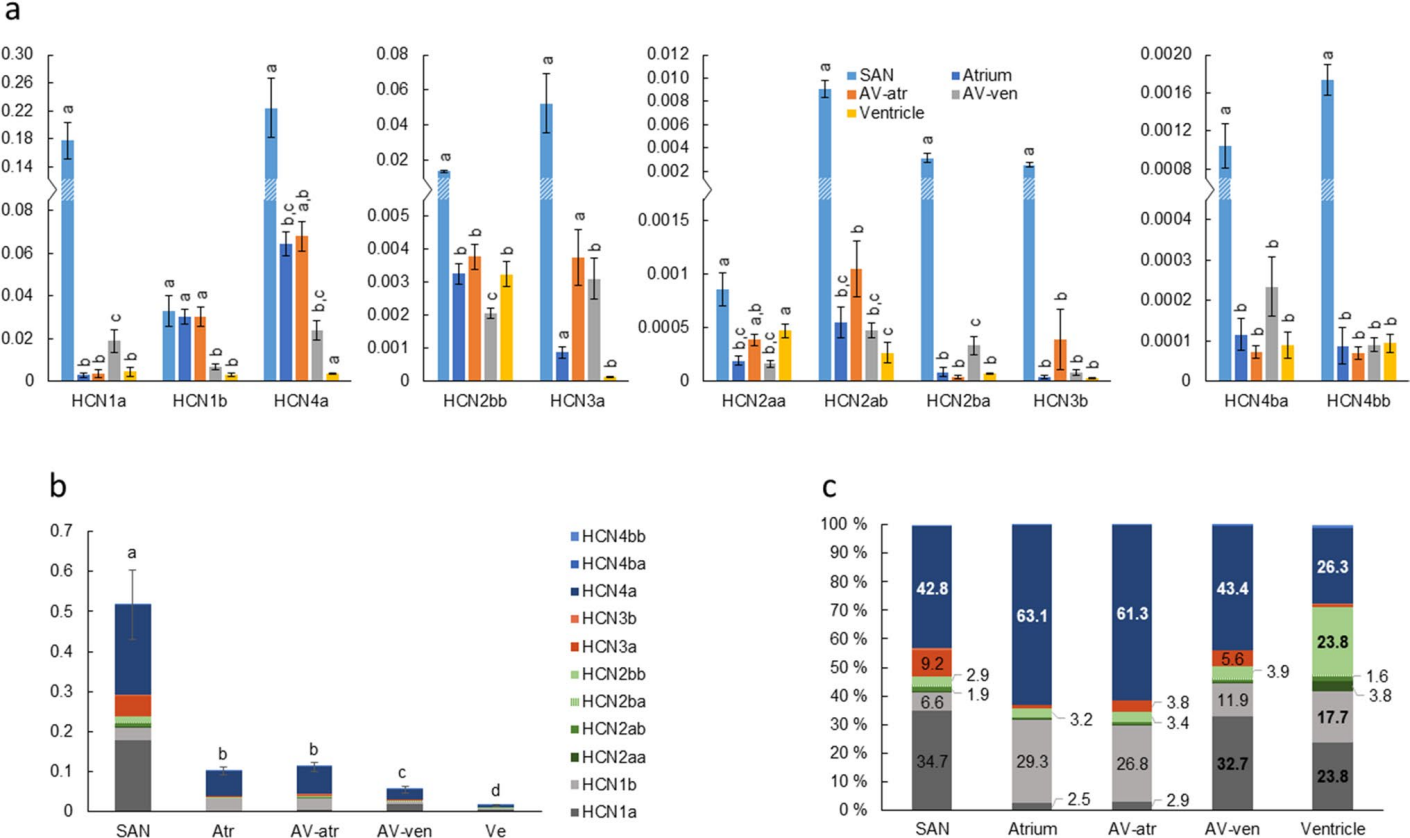
Pacemaker channels. **a** Transcript expression of HCN genes encoding pacemaker channels normalized to the expression of reference gene DnaJA2 (*n* = 5, mean ± S.E.M.). All known HCN isoforms of rainbow trout are included. Note the different *y*-axis scales of the diagrams. **b** Total transcript expression of HCN isoforms in the cardiac compartments. **c** Relative proportion (%) of each HCN isoform in cardiac compartments

Expression levels of all known rainbow trout HCN isoforms (HCN1a and -b, HCN2aa, -ab, -ba and -bb, HCN3a and -b, and HCN4a, -ba and -bb) were measured from different parts of the heart. HCN4a, -1a and -1b were the most expressed isoforms (Fig. 10). The most striking finding in HCN transcript expression was the higher abundance of HCN channels in SAN than in any other part of the heart (Fig. 10a). Total HCN transcript abundance decreased from caudal to cranial direction in the order: SAN >  > atrium = AV-atr > AV-ven > ventricle, as HCN transcripts in SAN were 5.1 ± 0.8-, 4.7 ± 0.8-, 9.3 ± 1.6- and 34.8 ± 5.8-fold compared to atrium, AV-atr, AV-ven and ventricle, respectively (Fig. 10b). With the exception of HCN1b and HCN2aa, all HCN isoforms were significantly more expressed in SAN than in other parts of the heart (Fig. 10a). No differences existed in HCN composition between AV-atr and atrium (*p* > 0.05). In contrast, HCN1a, -4a, -3a and -2ba as well as the total amount of HCN transcripts were higher in AV-ven than ventricle.

## Discussion

To our knowledge, this is the first study to investigate ion currents and ion channel expression in fish AV tissue. The trout AV canal forms a clear transition zone between the atrium and the ventricle in respect to ion channel composition and ion current densities. Yet, the AV nodal tissue has a typical electrophysiological phenotype that differs from those of SAN, atrium, and ventricle both in terms of ion current densities and expression of ion channel transcripts. In the rainbow trout AV canal, two types of AV cardiomyocytes, transitional cells and nodal cells, were distinguished: the transitional cells are intermediate between nodal cells and working atrial/ventricular myocytes, whereas the nodal cells are more similar to the SAN cells, because they have less negative *V*_rest_, small I_K1_ and no *I*_Na_. To which extent atrial and ventricular myocytes are an integral part of the fish AV canal, or represent contamination by actual atrial/ventricular tissue, remains to be elaborated by more detailed studies.

### Histology of the trout AV tissue

The histological structure of the AV region is preserved in adult teleost species and is characterized by a compact myocardium surrounded by the connective tissue (McWilliam 1885; Santer and Cobb 1972; Sedmera et al. 2003; Icardo and Colvee 2011; Stoyek et al. 2016). However, some variation in the amount of connective tissue and vascularization is observed between fish species (Icardo and Colvee 2011). In the heart of rainbow trout (and other teleosts), the AV region is composed of a myocardial ring or canal that is continuous with atrial and ventricular myocardia and laterally protected by robust connective tissue layers (Icardo and Colvee 2011). This arrangement of tissues is functionally relevant—to delay AP conduction—if atrial and ventricular myocytes are not in direct contact in any area of the canal. Therefore, we must assume that there is a zone composed exclusively of AV nodal myocytes that functionally separates atrial and ventricular myocardia. As the sole electrophysiological link between the atrium and the ventricle, the AV canal affects the function of the heart with its special structural and electrophysiological properties in normal physiological conditions and under environmental stresses. In particular, the small contact area between AV-ven and the ventricle proper is a potential site for AV block during acute heat stress. When a small cell/tissue mass (AV-ven) meets a much larger cell/tissue mass (ventricle), there is unfavorable ratio between depolarizing (source) current of active AV cells and repolarizing (sink) current of resting ventricular cells, which may prevent ventricular excitation (Vornanen 2020). In the zebrafish heart, the AV canal appears to be connected to the endocardial trabeculae by two specific tracts, whereas the contact of the AV canal with the outer compact myocardium seems to be blocked by an insulating wedge of connective tissue (Sedmera et al. 2003; Icardo and Colvee 2011). The role of the contact area between the AV canal and ventricular trabeculae in heat-induced ventricular bradycardia should be further investigated.

Although the morphology and histology of the fish AV area is fairly well known, the cell types that make up the myocardial tissue of the AV canal are less well elucidated (Santer and Cobb 1972; Sedmera et al. 2003; Icardo and Colvee 2011; Icardo 2017). In mammals, myocytes of the AV tissue are traditionally classified based on either their morphological or electrophysiological characteristics. Morphologically mammalian AV myocytes are described in three major categories: rod-shaped, ovoid and spindle-shaped cells (Anderson 1972; Munk et al. 1996; Ren et al. 2006). Three morphologically distinct myocyte types (star-, spider- and spindle-shaped cells) were identified in the rainbow trout AV tissue. However, based on electrophysiological properties, only two types of myocytes could be distinguished, which were classified as either nodal AV myocytes or transitional AV myocytes. The star- and spider-shaped myocytes were generally nodal AV myocytes in their electrophysiological characteristics, whereas the spindle-shaped myocytes could electrophysiologically be either nodal or transitional AV myocytes. In this respect, trout AV cells are similar to mammalian AV cells whose electrophysiological phenotype cannot be reliably determined from their cellular morphology (Munk et al. 1996; McGuire et al. 1996). Since electrophysiological properties of trout AV cells cannot be inferred from their light microscopic appearance, their classification must be based on electrophysiological properties.

### Electrophysiological properties of trout AV myocytes

Cardiomyocytes within the AV tissue are surrounded by considerable amount of connective tissue (Icardo and Colvee 2011), and therefore, isolation of AV cells requires a much longer digestion protocol than atrial and ventricular myocytes. Therefore, when isolating trout AV myocytes, the AV region was subjected to additional 20 min of enzymatic digestion after finishing the perfusion of the whole heart. Atrial and ventricular myocytes of the rainbow trout heart do not tolerate such long digestion period and are likely to die. Thus, we assume that nodal and transitional cells obtained from the AV region of the rainbow trout heart represent real AV myocytes, not potentially contaminating atrial or ventricular myocytes.

APs of the vertebrate AV node (AVN) are slowly rising and V_rest_ is less negative and AP amplitude smaller than in atrial and ventricular muscles (Hoffman et al. 1959; Billette 1987). The differences in AP shape are due to differences in ion channel composition and ion current characteristics of the nodal tissues (George et al. 2017). Moreover, the mammalian atrioventricular node (AVN) is divided into three regions based on the shape of AP: atrio-nodal (AN), nodal (N) and nodal-His (NH) cells (De Carvalho and De Almeida 1960). N cells are “real” nodal cells found in the compact node, whereas AN and NH myocytes are transitional cells located in the transitional zone and penetrating the bundle, respectively (Munk et al. 1996; Greener et al. 2009). The mammalian N cells are characterized by relatively depolarized *V*_rest_, diastolic depolarization, slow rate of AP upstroke and ability to spontaneous pacemaking, whereas AN and NH cells have intermediate properties between atrial and nodal myocytes, i.e., having a fast rate of AP upstroke and a stable, negative *V*_rest_ (Greener et al. 2009, 2011; Dobrzynski et al. 2013; Billette and Tadros 2019). Cardiomyocyte isolated from the rainbow trout AV region seemed to belong to either nodal AV myocytes or transitional AV myocytes, perhaps corresponding mammalian N and AN/NH cells, respectively. The trout nodal myocytes had a tiny I_K1_ which meant they were unable to maintain a negative V_rest_ typical of atrial and ventricular myocytes. Importantly, the highly depolarized value of V_rest_, measured with a current clamp in nodal cells, is an artifact due to the inward leakage current that makes the cell depolarized when I_K1_ is tiny. Further studies using sharp microelectrodes should find the true *V*_rest_ values typical of these cells. The nodal myocytes always lacked I_Na_ but had a robust I_Ca_ characterized by the peak current at about -20 mV and low sensitivity to nifedipine. Since the RT-PCR data showed a high expression level of Ca_V_3.1a (CACNA1Ga) transcripts, it can be assumed that this current is a T-type Ca^2+^ current (*I*_CaT_), although further experiments are needed to clarify this point. It should be noted, however, that also I_CaL_ was expressed in AV node cells. Indeed, the above characteristics of trout nodal cells resemble those of the mammalian N cells, where I_K1_ is small, I_Na_ is absent and AP upstroke is generated by *I*_CaL_/*I*_CaT_ (George et al. 2017). The most notable difference in electrophysiological properties between trout and mammalian AV nodal tissues is perhaps the total absence of I_f_ in the nodal cells of the trout heart. Unlike trout nodal cells, most mammalian N cells have the hyperpolarization-activated current (*I*_f_) (Noma et al. 1980; Munk et al. 1996). The electrophysiological properties of the trout AV nodal cells are suitable for slow AP conduction (absence of *I*_Na_) and pacemaking activity (depolarized *V*_rest_ and virtual absence of *I*_K1_), although the ionic basis of the pacemaking remains open in the absence of *I*_f_. Despite the distinct differences in morphological structure of the AV tissue between mammalian and fish hearts (bundle or node vs. canal), the cellular diversity and functional properties seem to be similar.

The transitional AV myocytes have a prominent *I*_K1_ and a stable negative *V*_rest_. Unlike the nodal cells, they have *I*_Na_ but lack *I*_CaT_. These features make transitional AV cells more like working ventricular myocytes but can be distinguished from them by a lower density of *I*_K_, *I*_K1_, and almost fourfold lower density of I_Na_. The most striking difference between transitional AV cells and working atrial myocytes is the higher *I*_K1_ density of the transitional AV cells. Therefore, transitional AV cells represent a specific type of fish cardiac myocytes, different from either nodal AV cells or working atrial and ventricular myocytes. The electrophysiological properties of trout transitional AV myocytes resemble those of mammalian AN cell where depolarization is achieved by *I*_Na_, *I*_f_ is absent and *I*_Ca_ is smaller than in N cells (Munk et al. 1996).

### Transcript expression of ion channel genes

Although the ion channel expression profile of the mammalian cardiac conduction system is quite well known (Schram et al. 2002a; Gaborit et al. 2007; Greener et al. 2009, 2011; Chandler et al. 2009; Atkinson et al. 2013), practically nothing is known about it in fish hearts. Here, we provide the first look at the ion channel diversity of AV nodal tissue of teleost heart. One characteristic of fish ion channels is the large number of gene paralogs which originates from the whole genome duplications in the teleost lineage (Jegla et al. 2009; Glasauer and Neuhauss 2014; Hassinen et al. 2019). The high diversity of ion channel genes provides raw material for adaptation to different environmental conditions (Glasauer and Neuhauss 2014).

Regional differences existed in the expression of ion channel transcripts between AV tissue and other cardiac compartments of the rainbow trout heart. 13 out of 42 genes studied were expressed differently between AV-ven region and the ventricle. In contrast, the transcript expression profile of the AV-atr was nearly identical with that of the atrium. Only 3 out of the 42 genes studied were significantly differently expressed between the atrium and the AV-atr: SCN4Aba showed lower, and CACNA1Ga and HCN3a higher mRNA expression in the AV-atr region than in the atrium. Thus, it is likely that the AV-atr sample was contaminated by atrial tissue. It should be noted, however, that vertebrate AVN is a heterogenous tissue and atrial myocytes are an integral part of the functional AV conduction system (George et al. 2017).

The relatively depolarized V_rest_ of mammalian N cells (see “Electrophysiological properties of trout AV myocytes”) is due to the total absence or low density of *I*_K1_ current, which in turn is due to the low mRNA expression level of the main I_K1_ producing channel Kir2.1 (Greener et al. 2009, 2011; Dobrzynski et al. 2013). Similarly, the expression of the main ventricular Kir2 channel of the trout heart, Kir2.4, was lower in the AV-ven region than in the ventricle. The Kir2 expression correlates well with the smaller I_K1_ and less negative *V*_rest_ of trout nodal cells. KCNH6/7 channels generate the I_Kr,_ the main repolarizing current of fish cardiac myocytes at AP plateau voltages, which regulate AP duration but are unlikely to be involved in maintaining *V*_rest_ (Vornanen et al. 2002; Galli et al. 2009; Haverinen and Vornanen 2009; Abramochkin and Vornanen 2015; Filatova et al. 2019). KCNH6/7 expression was highest in the atrium and lowest in the AV-ven which correlates reasonably well with I_K_ densities of atrial and AV nodal cells.

Although *I*_f_ current was not found in the cells of trout AV tissue, HCN channels were expressed to some extent in AV tissue (and all parts of the trout heart). Expression levels of the major HCN channels of the rainbow trout heart, HCN1a and HCN4a, were higher in the AV-ven region than in the ventricle consistently with the spontaneous activity of the trout AV canal (Abramochkin et al. unpublished) (Stoyek et al. 2016). Since I_f_ was not found in trout AV myocytes, I_f_ may play a minor role in the spontaneous activity of the trout AV canal. The present findings are consistent with the histochemical studies of zebrafish heart; some polygonal HCN4-positive cells are present in zebrafish AV region but in the absence of Islet-1, their identity as pacemaker cells remains open (Stoyek et al. 2016). In humans, rabbits and rats, the HCN4 channel is abundantly expressed in the AVN (Yoo et al. 2006; Greener et al. 2009, 2011). However, even in mammalian hearts, not all AVN myocytes express I_f_ (Noma et al. 1980; Munk et al. 1996; Nikmaram et al. 2008; Cheng et al. 2011). It is clear that more detailed studies are needed on the ionic mechanism of the nodal cells of the fish heart.

Slow rate of AP upstroke is typical for N cells of the mammalian AVN, which is due to low expression level of Na^+^ channels (Yoo et al. 2006). AP upstroke in mammalian AVN myocytes is achieved by I_CaL_ rather than I_Na_ (Dobrzynski et al. 2013). In terms of Na^+^ and Ca^2+^ channels/currents, the AV tissue of the trout heart is largely similar to the mammalian N cells. In trout, a small I_Na_ existed in transitional AV myocytes, whereas it was completely absent in nodal cells. In addition, the mRNA expression levels of Na^+^ channel isoforms SCN4Aba and SCN5LAba were lower in the AV-ven region than in the ventricle. In the human, AVN Ca_v_1.3 (CACNA1D) and Ca_v_3.1 (CACNA1G) are highly expressed (Greener et al. 2011). In the trout heart, Ca_v_3.1 was also more abundant in the AV-ven region than in the ventricle. In contrast, Ca_v_1.3 isoform was more weakly expressed in the AV-ven zone than in the working ventricular myocardium. The abundant expression of T-type Ca^2+^ channel genes correlates well with the electrophysiological data indicating the presence of two Ca^2+^ current types in nodal cells.

The vertebrate AVN acts as an auxiliary pacemaker if SAN function fails, albeit with a slower intrinsic rate (Meijler and Janse 1988; Stoyek et al. 2016; Billette and Tadros 2019). There are significant similarities in the ionic bases of AP formation between SA and AV tissue of the vertebrate heart, although ion currents of the AV tissue are not known as well as those of the SAN (Schram et al. 2002b; Marionneau et al. 2005; Greener et al. 2009). Therefore, trout AV canal and SAN might be expected to have some common electrophysiological features. Consistent with its putative role in cardiac pacemaking, the expression of HCN channels in the SAN was several-fold higher than in other parts of the trout heart. In the trout AV-atr, HCN expression was only about 20% of the SAN level. This difference in HCN channel expression between trout SA and AV tissue is very similar to that of the mouse heart (Marionneau et al. 2005). It should be noted, however, that although HCN channels are expressed in the SAN of the brown trout (*Salmo trutta*), the I_f_ current is very small and detected only in a minor subpopulation of SAN cells (Hassinen et al. 2017). The rate of AP upstroke in vertebrate nodal tissues is based on Ca^2+^ currents and is, therefore, much slower than in working atrial and ventricular myocytes (Irisawa 1978). Consistent with this Na^+^ channel expression in AV-ven was low, but quite surprisingly, Na^+^ channel transcripts in the SAN were much higher than in any other part of the trout heart. Sutcliffe et al. (Sutcliffe et al. 2020) also found a high transcript expression of Na^+^ channels in rainbow trout SAN. The abundance of Na^+^ channels is probably due to the abundant neuronal tissue which tightly surrounds the primary pacemaker of rainbow trout heart (Yamauchi and Burnstock 1968; Haverinen and Vornanen 2007). Islet-1 (ISL1) is a transcription factor involved in cardiogenesis and a marker of pacemaker cells in zebrafish SAN (Tessadori et al. 2012; Kelder et al. 2015; Stoyek et al. 2016). In rainbow trout heart, ISL1 isoforms were expressed mainly in SAN, with very low expression level (≤ 1.5% of the SAN level) in the AV canal and other parts of the heart. While this is consistent with its role in the fish SAN in supporting pacemaker activity via maintenance of the gene expression profile of the SAN cells, it may have little role in AV nodal tissue (Vedantham et al. 2015). Both L- and T-type Ca^2+^ channels were expressed in trout SAN, transcripts of CACNA1Daa, -Dab, -Dba, -Ga, -Gb and -Ha being more abundant in SAN than in atrium. This is in accordance with mammalian SAN where CACNA1D and CACNA1G are more abundant than in atrium (Chandler et al. 2009).

### Limitations and perspectives

Although the current study provides the first glimpse to the ionic function of the fish AV canal, more research is needed to specify the electrophysiological properties and ion channel composition of nodal and transitional myocytes. In particular, transcript expression of ion channels should be measured from pure AV myocyte samples collected by microdissection, or alternatively in situ hybridization should be used to localize gene expression. However, ion channels are often expressed at low levels which makes the in situ approach difficult. Connexins form gap junctions which have an important role in mammalian AVN function and deserve research in fish as well. This will be, however, a tedious task because there are at least 77 connexin genes in the rainbow trout genome (GenBank). However, further molecular studies and related functional experiments at the whole tissue level should be pursued to understand AV tissue function in thermal responses of the fish heart.

## Data Availability

Gene sequences can be found in GenBank.
